# G protein-coupled receptor kinases in hypertension: physiology, pathogenesis, and therapeutic targets

**DOI:** 10.1038/s41440-024-01763-y

**Published:** 2024-07-03

**Authors:** Fuwei Zhang, Ines Armando, Pedro A. Jose, Chunyu Zeng, Jian Yang

**Affiliations:** 1 Research Center for Metabolic and Cardiovascular Diseases, The Third Affiliated Hospital of Chongqing Medical University, Chongqing, PR China; 2 Department of Nutrition, The Third Affiliated Hospital of Chongqing Medical University, Chongqing, PR China; 3 Department of Cardiology, The Third Affiliated Hospital of Chongqing Medical University, Chongqing, PR China; 4 Division of Renal Diseases & Hypertension, Department of Medicine and Department of Physiology/Pharmacology, The George Washington University School of Medicine & Health Sciences, Washington, DC USA; 5 Department of Cardiology, Daping Hospital, The Third Military Medical University (Army Medical University), Chongqing, PR China; 6 Chongqing Key Laboratory for Hypertension Research, Chongqing Cardiovascular Clinical Research Center, Chongqing Institute of Cardiology, Chongqing, PR China

**Keywords:** Blood pressure, G protein-coupled receptor, G protein-coupled receptor kinase, Hypertension

## Abstract

G protein-coupled receptors (GPCRs) mediate cellular responses to a myriad of hormones and neurotransmitters that play vital roles in the regulation of physiological processes such as blood pressure. In organs such as the artery and kidney, hormones or neurotransmitters, such as angiotensin II (Ang II), dopamine, epinephrine, and norepinephrine exert their functions via their receptors, with the ultimate effect of keeping normal vascular reactivity, normal body sodium, and normal blood pressure. GPCR kinases (GRKs) exert their biological functions, by mediating the regulation of agonist-occupied GPCRs, non-GPCRs, or non-receptor substrates. In particular, increasing number of studies show that aberrant expression and activity of GRKs in the cardiovascular system and kidney inhibit or stimulate GPCRs (e.g., dopamine receptors, Ang II receptors, and α- and β-adrenergic receptors), resulting in hypertension. Current studies focus on the effect of selective GRK inhibitors in cardiovascular diseases, including hypertension. Moreover, genetic studies show that GRK gene variants are associated with essential hypertension, blood pressure response to antihypertensive medicines, and adverse cardiovascular outcomes of antihypertensive treatment. In this review, we present a comprehensive overview of GRK-mediated regulation of blood pressure, role of GRKs in the pathogenesis of hypertension, and highlight potential strategies for the treatment of hypertension.

**Figure:**
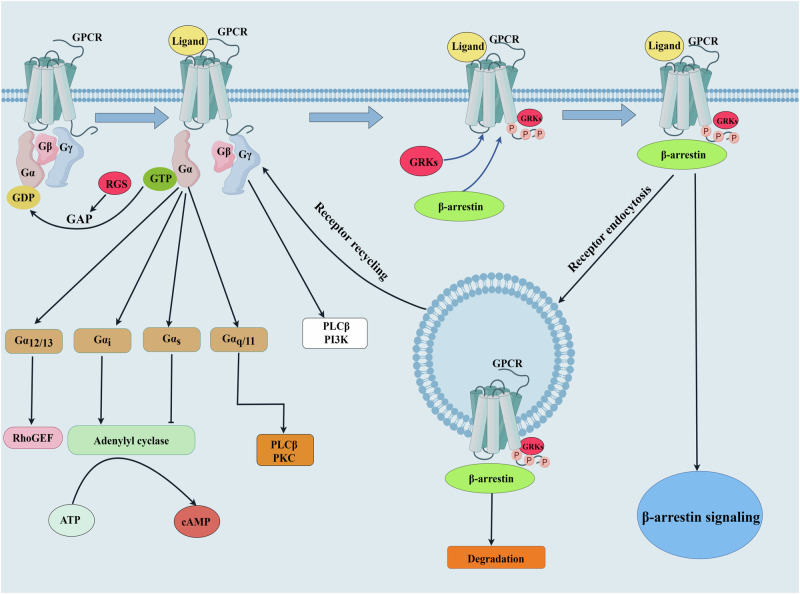
Schematic representation of GPCR desensitization process. Activation of GPCRs begins with the binding of an agonist to its corresponding receptor. Then G proteins activate downstream effectors that are mediated by various signaling pathways. GPCR signaling is halted by GRK-mediated receptor phosphorylation, which causes receptor internalization through β-arrestin.

Schematic representation of GPCR desensitization process. Activation of GPCRs begins with the binding of an agonist to its corresponding receptor. Then G proteins activate downstream effectors that are mediated by various signaling pathways. GPCR signaling is halted by GRK-mediated receptor phosphorylation, which causes receptor internalization through β-arrestin.

Essential hypertension, also known as primary hypertension, is a major contributing risk factor for cardiovascular and cerebrovascular diseases, and causes damage of target organs, such as the kidney, heart and brain [1]. Worldwide data from the Noncommunicable Disease (NCD) Risk Factor Collaboration show that the number of people 30–79 years old with hypertension has doubled from 1990 to 2019, with most of the increase occurring in low-income and middle-income regions [2]. In the United States, 46.7% (122.4 million) of adults ≥20 years of age has hypertension, and the number of deaths attributable to high blood pressure increased 90.1% from 2010 to 2020 [3]. Hypertension is a major public health problem and challenges worldwide [4].

Cells communicate with each other by receiving and sending through a myriad of extracellular proteins/molecules, such as neurotransmitters, chemoattractants, and hormones, which bind to receptors and drive the initiation of multiple intracellular signaling routes. G protein-coupled receptors (GPCRs) are the largest superfamily of cell-surface receptors and the most diverse group of proteins involved in transmembrane signal transduction [5, 6]. They share conserved seven α-helical structured transmembrane domains, extracellular ligand-binding domains, and carboxy-terminal intracellular domains. GPCRs, including adrenergic receptors, angiotensin II (Ang II) receptors, and dopamine receptors, control a variety of physiological processes involved in the regulation of blood pressure [7, 8]. Cell dysfunction, induced by aberrant GPCRs, including abnormal phosphorylation, trafficking, and expression, causes hypertension by various mechanisms [8–10].

The phosphorylation and desensitization of agonist-occupied GPCRs are primarily mediated by GPCR kinases (GRKs) [11]. As their name implies, GRKs are defined by their actions on GPCR phosphorylation, receptor internalization, and subsequent signal termination (Fig. 1). Abnormalities of GRKs are associated with cardiovascular diseases, including hypertension [12, 13]. A number of studies have shown that GRKs play a vital role in the regulation of blood pressure; aberrant expression and/or activity of GRKs cause hypertension and are associated with impaired response to antihypertensive treatment [14–17]. There are abundant epidemiological and animal studies reinforcing the role of GRKs and their underlying mechanisms in the regulation of blood pressure. Therefore, in this article, we review our evolving understanding of GRK-mediated regulation of blood pressure and highlight potential strategies for targeting GRKs in the prevention and treatment of hypertension.

**Fig. 1:**
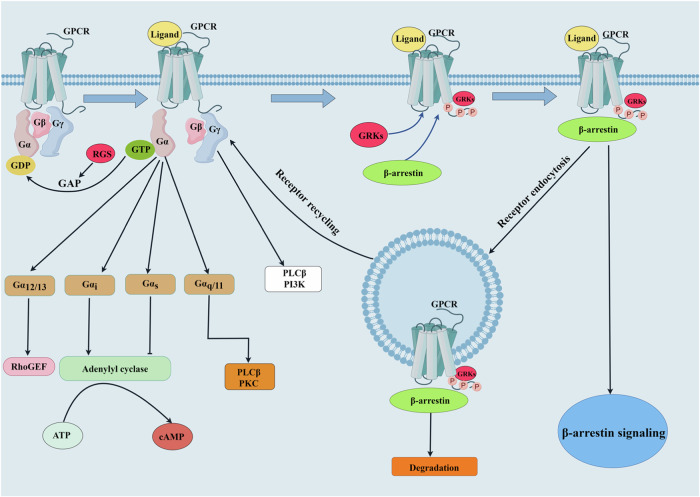
Schematic representation of GPCR desensitization process. Activation of GPCRs begins with the binding of an agonist to its corresponding receptor. Then G proteins activate downstream effectors that are mediated by various signaling pathways. GPCR signaling is halted by GRK-mediated receptor phosphorylation, which causes receptor internalization through β-arrestin. ATP adenosine triphosphate, cAMP cyclic adenosine monophosphate, GAP GTPase activating protein, GDP Guanosine-5’-diphosphate, GEF guanine nucleotide exchange factor; GPCR G protein-coupled receptor, GRK G protein–coupled receptor kinase, GTP guanosine triphosphate, PI3K phosphatidylinositol-3 kinase, PKC protein kinase C, PLC-β phospholipase C-β; RGS regulators of G protein signaling

## The Grk family

GRK-mediated receptor phosphorylation is one of the well-characterized mechanisms for GPCR desensitization. There are more than 800 distinct GPCRs in the human genome but of the vital regulators of agonist-induced phosphorylation of GPCRs, only seven GRKs (GRK1–7) have been identified [18]. Based on their sequence similarity and gene structure, the serine/threonine kinase GRK family encompasses seven protein isoforms that are classified into three subfamilies. GRK1 and GRK7 belong to the GRK1-like subfamily, also known as visual or rhodopsin-specific GRKs. GRK2 (β-adrenergic receptor kinase 1, β-ARK1) and GRK3 (β-ARK2) belong to the GRK2-like subfamily, also known as β-adrenergic receptor (β-AR) kinase subfamily. GRK4, GRK5, and GRK6 belong to the GRK4-like subfamily [17, 18].

The seven GRKs share a common general protein structure with a central catalytic domain (~270 amino acids), flanked by an amino-terminal (N-terminal) domain (~185 amino acids) and a carboxyl terminal (C- terminal) domain (~105 to 230 amino acids) [19]. The N-terminus, a well-conserved domain between GRK members, is vital for the selective recognition of the activated receptor and also contains a region termed as a regulator of G protein signaling homology (RH) domain (~120 amino acids). However, the C-terminus is divergent among GRK subfamilies and contributes to their subcellular membrane localization and agonist-dependent translocation [19, 20].

In addition to GPCRs, GRKs also regulate the phosphorylation or expression of other non-GPCR receptors, such as adiponectin receptor 1 (AdipoR1) and insulin-like growth factor-1 receptor [21, 22]. Moreover, GRKs regulate non-receptor substrates, including signaling proteins, such as insulin receptor substrate-1, nuclear proteins, such as histone deacetylase 5, and transcription factors, such as IκBα, among others [23–26]. We also reported that GRK4 increases the phosphorylation of signal transducer and activator of transcription 1 and HDAC4 [27, 28]. It should also be noted that kinase activity is not necessary for some GRK-mediated functions. For example, GRK4 regulates cilia and kidney development independent of its kinase function [29].

All GRKs are primarily localized in the cytosol and plasma membrane. However, the tissue distribution of the seven GRK subtypes is different from each other. GRK2, GRK3, GRK5, and GRK6 are ubiquitously distributed among mammalian tissues [17]. GRK1, GRK4, and GRK7 are expressed in specific tissues. GRK1 and GRK7 are expressed exclusively in the retina, whereas GRK4 is expressed in a few organs [17, 30]. These indicate that GRKs may exert different physiological functions based on their tissue distribution.

## Role of Grks on blood pressure regulation

**Fig. 2:**
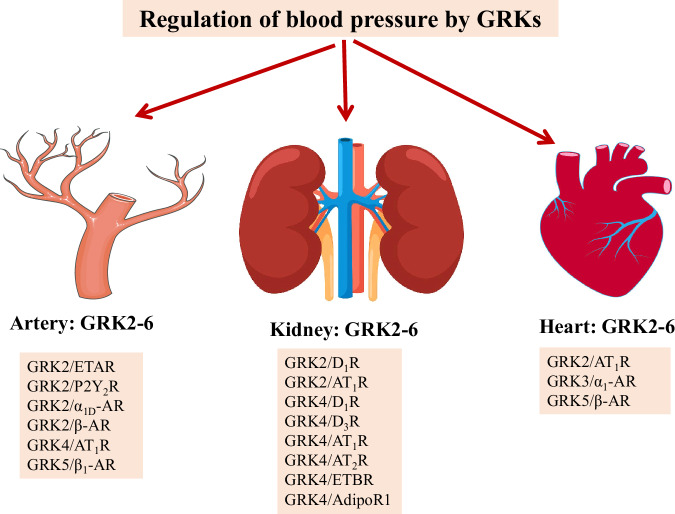
Regulation of blood pressure by GRKs. GRKs exert their functions in organs, including kidney, artery, and heart, by regulating the expression or phosphorylation of various GPCRs to modulate blood pressure. Pink color: various substrates regulated by GRKs. α-_1D_AR α-_1D_ adrenergic receptor, adipoR1 adiponectin receptor 1, Ang II angiotensin II, AT_1_R angiotensin II type 1 receptor, AT_2_R angiotensin II type 2 receptor, β-AR β-adrenergic receptor, D_1_R dopamine D_1_ receptor, D_3_R dopamine D_3_ receptor, ETBR endothelin receptor type B, GRK G protein-coupled receptor kinase, P2Y_2_R P2Y_2_ receptor

Many studies have shown that GRKs exert their biological functions by mediating the regulation of agonist-occupied GPCRs, non-GPCR receptors, and non-receptor substrates (Table 1). Several organs, including the artery, heart, and kidney, are involved in GRK-mediated blood pressure regulation (Fig. 2). The expression and/or activity of GRKs are aberrant in the hypertensive humans and animal models of hypertension. Because GRK1 and GRK7 are expressed only in the retina, only the role of the other five GRKs in the regulation of blood pressure have been studied.

**Table 1:** GRK substrates related to the regulation of blood pressure

GRK isoform	Substrate protein	Tissue/cells	Regulation of Substrate
GPCR substrates			
GRK2	ETAR	Rat mesenteric arterial smooth muscle cells [35]; rat aortic smooth muscle cells [37]	Inactive dominant-negative GRK2 or siRNA-*Grk2* attenuates ETAR desensitization and its mediated VSMC contractile or migration signaling [35, 37]
	P2Y_2_R	Rat mesenteric artery [36]; rat mesenteric arterial smooth muscle cells [36]; rat aortic smooth muscle cells [37]	GRK2 inhibition or siRNA-*Grk2* attenuates P2Y_2_R desensitization and its mediated VSMC contractile or migration signaling [36, 37]
	β-AR	Mouse aortic arterial smooth muscle cells [34]; brachial artery [66]; HEK293 cells [76]	VSM-targeted overexpression of *Grk2* attenuates β-AR signaling and vasodilation [34]; inhibition of GRK2 activity prevents β-AR desensitization [66]; GRK2 directly interacts with β_2_-AR and regulates its desensitization [76]; *Grk2* knockout or GRK2 inhibition increases β-AR-mediated vasodilation [39]
	AT_1_R	HEK293 cells [38]; kidney [43]; heart [32]	GRK2 inhibition reduces agonist-induced AT_1_R phosphorylation and prevents receptor desensitization [38]; *Grk2* knockdown enhances renal AT_1_R-mediated ROS production [43]; cardiac-specific *Grk2* expression attenuates Ang II-induced increase in cardiac contractility [32]
	D_1_R	Rat RPT cells [47, 49]; OK cells [48]; HEK293 cells [41, 75] and rat striatum [75]	GRK2 mediates H_2_O_2_ or insulin-caused renal D_1_R phosphorylation and impairs D_1_R-mediated inhibition of Na^+^-K^+^-ATPase activity [47, 48]; GRK2 increases D_1_R desensitization and phosphorylation [75]; *Grk2* overexpression increases agonist-induced D_1_R phosphorylation [41]
	α_1D_-AR	VSM	*Grk2* knockout or peptide inhibition enhances α_1D_-AR-induced vasoconstriction [39]
GRK3	α_1_-AR	Cardiac myocytes	GRK3 inhibition causes α_1_-AR hyper-responsiveness [73]
	D_1_R	HEK293 cells; rat striatum	GRK3 increases D_1_R desensitization and phosphorylation [75]; *Grk3* overexpression increases dopamine-induced D_1_R phosphorylation [41]
	β_2_-AR	HEK293 cells	GRK3 directly interacts with β_2_-AR and regulates its desensitization [76]
	AT_1_R	HEK293 cells	*Grk3* overexpression increases angiotensin II-induced AT_1_R phosphorylation [38]
GRK4	D_1_R	CHO cells [15]; HEK293T cells [85]; Human RPT cells[15, 86]; Kidney [81]; Rat renal cortex [79];	GRK4 phosphorylates the D_1_R, causing its constitutive desensitization and internalization [85]; GRK4 As-Odns decrease serine-phosphorylated D_1_R [79]; UTMD-mediated renal *Grk4* siRNA delivery reduces D_1_R phosphorylation and blood pressure and increases D_1_R-mediated natriuresis and diuresis [81]; *GRK4γ* variants increase D_1_R phosphorylation and impair D_1_R-mediated cAMP production [15]; GRK4 inhibition blunts D_1_R desensitization [86]
	D_3_R	Human RPT cells	GRK4γ and GRK4α increase the phosphorylation of agonist-activated D_3_R [91]
	AT_1_R	Thoracic aortic VSM cells [77]; HEK293 cells and human RPT cells [78]; kidney [78]	*hGRK4γ 142V* increases AT_1_R expression [77, 78]
	ETBR	Renal cortex; rat RPT cells	*hGRK4γ 142V* increases but GRK4 depletion decreases renal ETBR phosphorylation [82]
	AT_2_R	Renal cortex; rat RPT cells	*hGRK4γ 142V* increases renal AT_2_R phosphorylation and causes its dysfunction [93]
	CCKBR	Renal cortex; rat RPT cells	*hGRK4γ 142V* increases but GRK4 silencing decreases renal CCKBR phosphorylation [170]
GRK5	AT_1_R	HEK293 cells	*Grk5* overexpression increases the angiotensin II-induced AT_1_R phosphorylation [38]
	β-AR	VSM [122]; heart [32]	VSM-*Grk5* over-expression increases blood pressure to a greater extent in male than female mice; β-AR stimulation induced vasoconstriction in male mice but decreased β-AR-induced vasodilation and increased angiotensin II sensitivity in female mice [122]; Cardiac-specific *Grk5* expression increases β-AR desensitization but does not affect the contractile response to angiotensin II [32]
	D_1_R	HEK293 cells	*Grk5* overexpression increases agonist-induced D_1_R phosphorylation [41]
GRK6	D_1_R	Intestinal epithelial cells	GRK6 antibodies prevents the desensitization-mediated loss of D_1_R inhibition of Cl/HCO3^-^ exchanger activity [130]
Non-GPCR receptor substrates			
GRK4	adipoR1	Renal cortex; rat RPT cells	hGRK4γ 142V causes adipoR1 phosphorylation and desensitization [21]
Non-receptor substrates			
GRK2	ENaC	Salivary duct cells	GRK2 phosphorylates S633 in the C terminus of ENaC [44]
	Nedd4 and Nedd4-2	HEK293T cells	GRK2 phosphorylates Nedd4 and Nedd4-2 [46]
GRK4	HDAC4	Mouse cardiomyocytes	GRK4 increases HDAC4 phosphorylation and decreases its binding to the beclin-1 promoter [28]
	NF-κB	Embryonic rat thoracic aortic smooth muscle cells	GRK4γ 142V increases NF-κB activity with more NF-κB bound to the AT_1_R promoter [77]
	STAT1	HK-2 cells and mouse kidney	GRK4 phosphorylates STAT1 [27]
	HDAC1	Human RPT cells and mouse kidney	GRK4γ 142V phosphorylates HDAC1 and promotes its nuclear export to the cytoplasm [78]

*α-AR* α-adrenergic receptor, adipoR1 adiponectin receptor 1, *As-Odn* antisense oligodeoxynucleotides, *AT*_*1*_*R* angiotensin II type 1 receptor, *AT*_*2*_*R* angiotensin II type 2 receptor, *β-AR* β-adrenergic receptor, cAMP cyclic adenosine monophosphate, *CCKBR* cholecystokinin receptor type B, *CHO* Chinese hamster ovary, *D*_*1*_*R* dopamine D_1_ receptor, *ENaC* epithelial sodium channel, *ETAR* endothelin receptor type A, *ETBR* endothelin receptor type B, *GRK* G protein-coupled receptor kinase, *HDAC1* Histone deacetylase 1, *HDAC4* Histone deacetylase 4, *hGRK4γ* human G protein-coupled receptor kinase 4-gamma, *Nedd4* neural precursor cell expressed developmentally downregulated 4, *NF-κB* nuclear factor-κB, *P2Y*_*2*_*R* P2Y_2_ receptor, *ROS* reactive oxygen species, *STAT1* signal transducer and activator of transcription 1, *VSM* vascular smooth muscle, *VSMC* vascular smooth muscle cell, *UTMD* ultrasound-targeted microbubble destruction

## GRK2

### Role of GRK2 in the regulation of blood pressure

GRK2 is ubiquitously expressed in mammalian organs and tissues, including the artery, heart, and kidney. GRK2 is not only a key regulator of vascular responsivity in resistance arteries but also regulates myocardial contractility [31, 32], and renal function (vide infra).

GRK2 regulates GPCR-mediated vascular signaling, which participates in the regulation of blood pressure. Global knockdown of GRK2 [33] or vascular smooth muscle (VSM)-targeted overexpression of GRK2 in mice increases resting blood pressure [34]. This apparent inconsistent effect on blood pressure may be related to the fact that the alteration in GRK2 expression (increased or decreased) can lead to the imbalance of vasoconstrictor and vasodilator effects, resulting in hypertension [33]. GRK2 negatively regulates GPCRs such as endothelin type A receptor [35], P2Y2 receptor [36, 37], angiotensin II type 1 receptor (AT_1_R) [38] and α_1D_-adrenergic receptor [39], which should result in vasodilation. By contrast, GRK2 also negatively regulates β_2_-adrenergic receptor [40] and dopamine D_1_ receptor (D_1_R)/adenylyl cyclase signaling [41], which should result in vasoconstriction. The rebound hypertension in mice that occurs after withdrawal of clonidine, an α_1D,_ α_2A-C_ adrenergic receptor agonist, is mitigated by an increase in nitric oxide bioavailability, an effect that is prevented by suppression of GRK2 activity [42]. However, one group reported that global GRK2 knockdown impaired kidney development, and increased blood pressure [43] while another group reported that *Grk2*^*+/−*^ mice have normal blood pressure and attenuated response to the hypertensive effect of Ang II infusion [31]. Therefore, the overall effect of GRK2 on blood pressure is difficult to predict (vide infra).

GRK2 directly interacts with the epithelial sodium channel (ENaC) [44], which increases the reabsorption of filtered sodium across the luminal membrane of the principal cells of the aldosterone-sensitive distal nephron [45]. GRK2 positively regulates ENaC activity by phosphorylating Nedd4 and Nedd4-2, two ubiquitin-protein ligases binding with the channels, to prevent their ability to inhibit epithelial channel activity [46]. The dopaminergic system plays a vital role in the regulation of sodium excretion. The activation of GRK2 by some factors such as oxidative stress and insulin impairs the ability of renal D_1_R to inhibit sodium transport [47, 48] that leads to the development of hypertension [8]. We have also reported that exposure to maternal diabetes mellitus causes renal D_1_R dysfunction and hypertension in adult rat offspring by increasing GRK2 activity [49]. Thus, GRK2 can increase blood pressure by increasing renal sodium transport.

GRK2 also regulates receptor-mediated signaling in the heart. GRK2 phosphorylates cardiac β_2_-adrenergic receptor and increases G_i_-biased signaling, which contributes to heart failure [50]. However, GRK2 inhibition increases β-adrenergic receptor-dependent cardiomyocyte and cardiac contractility and reverses cardiac dysfunction in heart failure models [51]. GRK2 also regulates the desensitization of other cardiac receptors, such as AdipoR1 and κ-opioid receptor [52, 53]. In addition, GRK2 participates in the regulation of other physiological or pathological functions in the heart, including fatty acid metabolism, production of NLRP3 inflammasome and oxidative stress, and modulation of insulin signaling [54–56]. How, these GRK2-mediated effects in the heart regulate blood pressure need to be studied further.

### Blood pressure phenotypes in mice with modified *Grk2* expression

As aforementioned, the role of GRK2 on the regulation of blood pressure has been demonstrated in mice with genetically modified *Grk2* expression. Since germline deletion of *Grk2* is lethal, *Grk2* hemizygous mice (GRK2^+/−^), shRNA-GRK2 transgenic mice, or knockdown in local organs have been used to investigate its effects on the regulation of blood pressure. shRNA-induced total body knockdown of GRK2 causes spontaneous hypertension and impairs vascular reactivity [33]. This is similar to the findings of another study in which *Grk2* knockdown in mice exacerbates kidney injury and causes spontaneous hypertension, accompanied with over-activation of the renal renin-angiotensin system (RAS) and AT_1_R signaling [43]. However, *Grk2* deficiency in global adult hemizygous mice does not cause hypertension but impairs the increase in blood pressure and prevents vascular remodeling secondary to Ang II infusion [31]. By contrast, inhibition of VSM-*Grk2* by overexpression of the C-terminal portion of *Grk2* or VSM-specific ablation of *Grk2* protein expression in mice has no effect on the increased blood pressure in the two-kidney, one-clip (2K1C) model, which may be due to a balance between the increased β-AR-mediated dilation and the enhancement of α_1D_-AR-stimulated vasoconstriction [39].

The reason for the inconsistent reports on the effect of *Grk2* knockdown on blood pressure remains unclear. We speculate some possibilities: 1) Different methods of *Grk2* knockdown may produce different effects. Global knockdown of GRK2 using a small hairpin (sh) RNA leads to hypertension [33, 43], a partial deficiency of *GRK2* protects against Ang II-induced hypertension [31]; VSM-specific *Grk2* knockdown has no effect on blood pressure [39]. 2) GRK2 regulates both vasoconstriction and dilation. For example, *GRK2* deficiency increases phenylephrine, but not KCl-mediated constriction and isoproterenol-mediated vasodilation [33]. The dominant effect induced by *GRK2* knockdown may determine the physiological or pathological phenotype. 3) Duration of *Grk2* silencing. There is a balance between vasoconstriction and vasodilation in *Grk2* knockdown mice. Twelve to 14 weeks of *Grk2* knockdown using small hairpin RNA causes a shift toward vasoconstriction and subsequent hypertension [33]. Additionally, activated renal AT_1_R-mediated signaling also plays an important role in the development of hypertension after six months of *Grk2* knockdown [43]. However, a longer (nine months) duration of *Grk2* knockdown was reported to lead to a nitric oxide-dependent vasodilation which overcomes vasoconstriction, making *Grk2*^*+/−*^ mice resistant to the development of hypertension [31]. 4) It is possible that the different effects on blood pressure induced by *Grk2* knockdown may be also attributed to experimental conditions such as animal species and shRNA induction.

The depletion of *Grk2* expression in aortic smooth muscle cells also impairs the proliferative effect of endothelin and Ang II [57], that should prevent the increase in blood pressure. *Grk2* knockdown in the endothelial cells of mesenteric arterioles promotes insulin-induced vasodilation and insulin-stimulated signaling pathway in SHRs; this effect is prevented by upregulation of *Grk2* [58]. Selective deletion of *Grk2* in vascular endothelium of mice also blunts the vasoconstriction induced by the α_1_-adrenergic receptor agonist phenylephrine and other vasoconstrictors (serotonin, oxytocin, and KCl) [59].

By contrast, transgenic mice with VSM-targeted *Grk2* over-expression have higher resting blood pressure than their wild-type littermates [34]. This is consistent with another study, which found that VSM *Grk2* over-expression leads to increased systolic blood pressure (SBP). Moreover, epinephrine injection increased SBP to a greater extent in the VSM-*Grk2* over-expressing mice than wild-type control mice [60]. However, cardiac-specific *Grk2* expression attenuates Ang II-induced increase in contractility and blood pressure [32], consistent with the pro- and anti-hypertensive effects of GRK2 that are tissue-dependent.

### GRK2 expression is increased in animal models of hypertension

The expression of *Grk2* mRNA is higher in the aorta but lower in the kidney of spontaneously hypertensive rats (SHRs) than Wistar-Kyoto (WKY) rats [61, 62]. GRK2 protein expressions in lymphocytes and VSMs are also higher in SHRs than Wistar- and WKY rats; vascular GRK2 protein expression is also higher in hypertensive Dahl salt-sensitive than normotensive Dahl salt-resistant rats [63]. The expressions of GRK2 and adrenergic receptors are decreased in the aorta but not in mesenteric arteries of NG-nitro-L-arginine methyl ester (L-NAME)-induced hypertensive rats, that is accompanied by decreased α_1_-adrenergic receptor-induced vasoconstriction along with increased β_2_-adrenergic receptor-mediated vasodilation; these changes are the opposite to those found in the aortas of SHRs [64]. In addition, renal GRK2 expression is increased in L-NAME-induced hypertensive rats. However, renal but not left ventricular GRK2 expression is decreased in SHRs [62].

### GRK2 and human hypertension

Studies have shown abnormal GRK2 expression and/or activity in the hypertensive state. GRK activity and GRK2 expression in lymphocytes are increased in hypertensive relative to normotensive humans [14]. The increase in GRK2 protein expression in lymphocytes from hypertensive subjects is positively associated with blood pressure and inversely associated with β-adrenergic agonist-mediated increase in adenylyl cyclase activity [14, 65]. Izzo et al. also reported that GRK2 expression is increased in blood lymphocytes of hypertensive patients and the impaired β-AR-mediated vasorelaxation in the brachial artery is restored by treatment with heparin, a nonspecific GRK inhibitor [66]. Further studies showed that in Black Americans, GRK2 but not GRK5 protein expression is increased 2-fold and GRK activity is increased >40% in the lymphocytes, which correlated with increasing blood pressure [67]. A case-control study in Han Chinese showed that the dominant model (CC vs. CT + TT) of rs1894111 polymorphism in the *ADRBK1* (*GRK2*) gene is associated with low-renin hypertension [68]. In addition, abnormal GRK2 expression or activity is associated with other types of hypertension. For example, elevated GRK2 levels in the umbilical vasculature are associated with elevated blood pressure in subjects with gestational hypertension and preeclampsia [69], but this may be a compensatory rather than a causative effect. Insufficient GRK2 activity compromises spiral artery remodeling and initiates necrotic events in the placenta, thereby causing preeclampsia [70].

## GRK3

GRK3 is ubiquitously expressed in mammalian organs and tissues. There are limited studies on the association between GRK3 and hypertension. GRK3 expression in cardiac myocytes progressively increases in 6-, 12-, and 24-month-old spontaneously hypertensive heart failure rats and is significantly higher than in control rats [71]. *Grk3* mRNA levels in human lymphocytes are inversely associated with SBP and diastolic blood pressure (DBP) [72], suggesting that GRK3 may play a beneficial role in the regulation of blood pressure. This is, at least in part, supported by findings in transgenic mice. Cardiac myocyte-restricted expression of the carboxyl-terminal fragment of *Grk3*, a competitive inhibitor of *Grk3*, results in a phenotype with elevated SBP and DBP but without alterations of heart rate in conscious, unrestrained mice [73]. Further studies found that the cardiomyocyte-restricted inhibition of *Grk3* in mice does not alter the hypertrophic response but attenuates cardiac dysfunction and heart failure with chronic pressure overload [74]. It should be noted that some GPCRs that regulate blood pressure, such as dopamine receptors and β-ARs are also regulated by GRK3 [75, 76]. *Grk3* expressed in HEK293 cells augments the agonist-induced AT_1_R or D_1_R phosphorylation, causing their desensitization [41]. However, the physiological consequences of altered GRK3 expression is unclear. Renal GRK3 expression is not different between WKY rats and SHRs but renal GRK3 mRNA is increased in the kidneys of rats made hypertensive with L-NAME [62]. The role of GRK3 in the regulation of blood pressure needs to be studied further.

## GRK4

### Effect of GRK4 on blood pressure

GRK4 is expressed to a greater extent in the testes and myometrium and to a lesser extent in other tissues such as the brain and kidney [13]. We also reported its expression in the artery and heart [28, 77], and found that renal and cardiac GRK4 levels are elevated by kidney ischemia-reperfusion injury and myocardial infarction, respectively [27, 28].

The role of GRK4 in the regulation of blood pressure is confirmed in several animal models of hypertension. Global *Grk4* knockout mice have lower SBP and DBP than *Grk4* wild-type (WT) mice [78]. Selective renal *Grk4* knockdown, via chronic renal cortical interstitial-selective infusion of *Grk4* antisense oligodeoxynucleotides, increases urine flow and ameliorates the sodium retention and elevated blood pressure of SHRs [79, 80]. We have reported that down-regulation of renal GRK4 expression, via ultrasound-targeted microbubble destruction-delivered *Grk4* small interfering RNA (siRNA), decreases blood pressure, accompanied with increased sodium excretion in SHRs [21, 81].

In contrast to the lowering of blood pressure caused by the silencing of *Grk4* [78–82], the over-expression of human *Grk4* gene variants (*hGrk4γ 65L*, *hGrk4γ 142V*, or *hGrk4γ 486V*) in mice has the opposite effect on blood pressure. On normal salt diet, transgenic mice with over-expression of human *GRK4γ* (*hGRK4γ*) variant *142V* have higher blood pressure than WT transgenic mice [15, 77, 78, 82]. However, *hGrk4γ 486V* [83] and *hGrk4γ 65L* transgenic mice have normal blood pressure on a normal-salt diet but have elevated blood pressure on a high-salt diet, indicating that *hGRK4γ 486V* and *hGRK4γ 65L* transgenic mice have salt-sensitive hypertension while *hGrk4γ 142V* transgenic mice have salt-resistant hypertension.

The reason why different GRK4 variants exhibit different phenotypes in blood pressure regulation is still unknown. Our previous study found that the salt sensitivity of *hGRK4γ 486V* mice is due, in part, to increased renal reactive oxygen species production, which can be attributed to defective renal antioxidant mechanisms [83]. However, the main reason for the different phenotypes of different GRK4 variants could be due to the different structures of the *GRK4* variants. We presume that the polymorphisms alter the structure of GRK4, that modulate its kinase activity, and affect substrate-regulated biological responses that subsequently cause hypertension. Allen et al. reported the overall crystal structure of GRK4α A486V and differences in the structure of GRK4α A486V from other GRKs. They found that relative to WT GRK4α, GRK4α A486V has an increased rate of autophosphorylation of a number of residues, which is due to a defective lag in the absence of preincubation with ATP [84]. However, the mechanism by which different *GRK4* polymorphisms affect enzymatic activity needs more investigation in future studies.

The control of blood pressure by GRK4 is due in part to its regulation of renal dopamine receptors. In human renal proximal tubule cells, GRK4 constitutively phosphorylates the D_1_R, even in the absence of agonist activation, causing its internalization and constitutive desensitization [85]. Treatment with heparin, an inhibitor of GRK activity, or antisense oligonucleotides (GRK4 > GRK2) blunt the agonist-induced D_1_R desensitization [86]. In human essential hypertension, single nucleotide polymorphisms (SNPs) in *GRK4γ* increase GRK activity and D_1_R serine phosphorylation, causing the uncoupling of D_1_R from its G protein/effector enzyme complex in the renal proximal tubule [15]. GRK4-mediated regulation of D_1_R expression or activity has been confirmed in several animal models of hypertension. We have reported that GKR4 impairs renal tubular and arterial D_1_R expression and D_1_R-mediated sodium excretion and vasodilation in offspring with hypertension related to in utero exposure to some factors such as fine particulate matter, e.g., PM_2.5_, lipopolysaccharide, and cold stress [87–89]. The impairment of the D_1_R function as part of the insulin resistance in type 2 diabetes mellitus in mice has also been related to increased GRK4 expression [90]. GRK4 also regulates the phosphorylation and function of the dopamine D_3_ receptor (D_3_R) in human proximal tubule cells [91]. However, the role of GRK4 in D_3_R-mediated regulation of blood pressure remains unclear.

GRK4 can also control blood pressure by interacting with the AT_1_R. *hGrk4γ 142V* phosphorylates HDAC1 and promotes its export from the nucleus to the cytoplasm resulting in an increase in the transcription of AT_1_R, presumably by decreasing its deacetylation [78]. AT_1_R blockade or the deletion of the *Agtr1a* gene normalizes hypertension in *Grk4γ 142V* mice [78]. We have reported that GRK4 is expressed in VSMCs of the aorta. AT_1_R expression is higher in the aorta of *Grk4γ 142V* transgenic mice than WT mice. Ang II-mediated vasoconstriction of the aorta is greater in *Grk4γ 142V* mice than in WT mice [77]. GRK4-positive regulation of AT_1_R expression and function is also found in other hypertensive animal models, including the male hypertensive offspring of male Sprague-Dawley rats that were exposed to fine particulate matter with an aerodynamic diameter of ≤2.5 μm [92]. We have also recently reported that GRK4 increases the phosphorylation of angiotensin II type 2 receptor (AT_2_R) which impairs renal AT_2_R-mediated diuresis and natriuresis, which participate in the GRK4-mediated regulation of blood pressure [93].In addition to dopamine and Ang II receptors, GRK4 also regulates other receptors such as the endothelin type B receptor (ETBR) and AdipoR1. Compared with WT mice, *hGRK4γ 142V* mice have increased phosphorylation of renal ETBR and AdipoR1 and impaired ETBR- and AdipoR1-induced diuresis and natriuresis [21, 82]. By contrast, renal-selective *GRK4* knockdown normalizes the increased ETBR and AdipoR1 phosphorylation, and partially or fully restores the ETBR- and AdipoR1-mediated increase in sodium excretion and reduction of blood pressure in SHRs [21, 82].

### GRK4 in hypertensive animal models

Basal GRK4 expression and activity in the kidney and artery are higher in SHRs than in WKY rats [61, 79, 80, 82, 93]. The increased GRK4 expression in the renal and artery in SHRs is organ-specific because there is no difference in cardiac GRK4 expression between WKY and SHRs [79]. Similar results are found in kidneys of humans with essential hypertension. Moreover, the increased activity of GRK4 in the kidneys of hypertensive subjects is caused not by increased renal GRK4 protein expression but rather by constitutively active variants of GRK4 [15]. The role of GRK4 in hypertension is further confirmed by studies involving renal *Grk4* depletion, which efficiently reduces GRK4 expression and lowers blood pressure in SHRs, accompanied with increased urine volume and sodium excretion [21, 79–82]. In addition, GRK4 expression and activity in the kidney and artery are increased in other animal models of hypertension [87–89, 94–96].

### GRK4 in human essential hypertension

The *GRK4* locus on human chromosome 4p16.3 is associated with essential hypertension and salt sensitivity [13]. Several *GRK4* gene variants, e.g., *R65L, A142V, and A486V*, are positively associated with essential hypertension in several ethnic populations [97–110] [Table 2]. However, the association between the *GRK4* variants and hypertension was not found in other studies, which may be age/sex/race/ethnicity-related, or a failure to study all the *GRK4* variants [103, 109, 111–114]. It should be noted that recent studies reported that some new *GRK4* variants are associated with hypertension (*GRK4 rs1644731*), both hypertension and diabetes (*GRK4 rs1557213*), or cardiovascular disease risk and diabetes (*GRK4 rs60314379*) [104, 105, 115]. In addition, an earlier meta-analysis showed that *GRK4 rs1024323* is associated with hypertension in Caucasians, but not in East Asians and Africans [106].

**Table 2:** GRK4 variants in hypertensive and normotensive subjects

*GRK4* Variant	Ethnic Group	Single or Multilocus Analyses	Clinical Findings
A142V (rs1024323)	Japanese	Single-locus	*Correlates with* low-renin hypertension [99]
	Japanese	Two-locus	*The combination of GRK4 A142V and CYP11B2* predicts low-renin essential hypertension [99]
	Japanese	Multi-locus	*The combination of GRK4 A142V, R65L*, and *A486V* predicts salt-sensitive hypertension [99]
	African- American	Single-locus	*Correlates* with blood pressure response to metoprolol among hypertensive male subjects [111]
	Black subjects	Single-locus	Predicts blood pressure response to dietary modification in Black subjects with mild-to-moderate hypertension [169]
	American	Single-locus	*Correlates* with increased risk for adverse cardiovascular outcomes [164]
	Japanese	Single-locus	*Correlates with hypertension; Carriers of hGRK4 142V have a greater decrease in SBP in response to ARBs* [165]
	Chinese	Single-locus	Hypertensive subjects with *GRK4 A142V* are more likely to be non-dippers [166]
A486V (rs1801058)	Italian	Single-locus	Associates with mild hypertension [102]
	Euro-Australian	Single-locus	*Correlates with hypertension* [101]
	Chinese	Single-locus	Associates with mild hypertension [100]
	Chinese	Single-locus	*Correlates with hypertension* [171]
	African American	Single-locus	Negatively *correlates* with hypertension in nonobese subjects [112]
	European	Single-locus	*Correlates* with salt sensitivity of blood pressure [107]
	African Brazilian	Two-locus	*NOS3-GRK4* interaction is related to DBP levels [98]
	American	Single-locus	*Correlates* with increased risk for adverse cardiovascular outcomes [164]
	Japanese	Single-locus	*Correlates with hypertension; Carriers of hGRK4 486V were less to achieve the blood pressure goal in response to an ARB* [165]
	Korean	Single-locus	A high sodium intake increases the obesity risk in children with *GRK4 A486V* regardless of sex [116]
	Korean	Multi-locus	A high sodium intake increases the obesity risk in boys with the *combination of GRK4 A486V*, *ACE*, and *SLC12A3* variants [116]
	Korean	Two-locus	*The combination of GRK4 A486V* and *CYP11B2* variants increases the obesity risk in girls when sodium intake is increased [116]
	Chinese	Single-locus	*H*ypertensive subjects with *GRK4 A486V* are more likely to be non-dippers [166]
R65L (rs2960306)	Ghanaian	Two-locus	*The combination of GRK4 R65L* and *ACE* predicts the hypertensive phenotype [108]
	American	Single-locus	Associates with stress-induced reduction in urinary sodium excretion in Black normotensive adolescents [117]
	Black subject	Single-locus	Predicts positive blood pressure response to dietary modification in Black subjects with mild-to-moderate hypertension [169]
	American	Single-locus	*Correlates* with increased risk for adverse cardiovascular outcomes [164]
	Swiss	Two-locus	Patients with *GRK4 65L* and *142* *V* need more antihypertensive treatment to reach the same MAP than homozygous carriers of only 1 variant or heterozygous/wild-type carriers of R65L, A142V, and A486V alleles [168]
	Korean	Single-locus	The *GRK4 65* *L* TT genotype is inversely correlated with hypertension risk [103]
	Chinese	Single-locus	Hypertensive subjects with *GRK4 R65L* are more likely to be non-dippers [166]
Other *GRK4* variants			
rs2488815	Chinese	Single-locus	Associates with the decreased eGFR [172]
rs1419044	American	Single-locus	Correlates with bevacizumab-induced hypertension [173]
rs1557213	Chinese	Single-locus	Contributes to the risk of hypertension [105]
rs1644731	Chinese	Two-locus	*GRK4 rs1644731* and *RDH8 rs1801058* are associated with hypertension [104]

*ACE* angiotensin-converting enzyme, *ARBs* angiotensin receptor blockers, *DBP* diastolic blood pressure, *eGFR* estimated glomerular filtration rate, *GRK4* G protein-coupled receptor kinase 4, *MBP* mean arterial blood pressure, *NOS3* nitric oxide synthase 3, *RDH8* retinol dehydrogenase 8, *SBP* systolic blood pressure, *SLC12A3* solute carrier family 12 member 3

*GRK4* variants are also associated with salt sensitivity. In a Japanese cohort, the genotype and allele frequencies of *GRK4* SNPs, including *R65L*, *A142V*, and *A486V*, are higher in salt-sensitive than in salt-resistant hypertensive subjects. Sodium excretion was reported to be inversely related to the number of *GRK4* variants in hypertensive persons, and the natriuretic response to dopaminergic stimulation is impaired in normotensive persons having three or more *GRK4* gene variants [99]. A large genome-wide association study in 15,034 Korean adults investigated the correlation of 1282 candidate SNPs with dietary sodium intake and found that the *GRK4 R65L* TT genotype is inversely associated with salt-sensitive hypertension risk [103]. Moreover, *GRK4* polymorphisms are also associated with salt sensitivity in normotensive subjects [107, 116]. The *GRK4 65L* allele is associated with impaired renal sodium handling in response to stress in Black normotensive adolescents [117].

## GRK5

GRK5 is ubiquitously expressed in mammalian organs and tissues, including the heart, lung, and retina [118]. GRK5 is involved in cardiovascular diseases, including cardiac fibrosis, heart failure, and hypertension [12, 119–124]. GRK5 mRNA and protein levels are increased in the aorta of Ang II- and norepinephrine-induced hypertensive rats [121]. *Gkr5* mRNA expression is also increased in the left ventricle and kidney of rats with hypertension induced by L-NAME [62]. However, in this report, the mRNA levels of renal GRK3 and GRK5 are not different between WKY and SHRs [62].

The role of GRK5 in the regulation of blood pressure is verified in transgenic mice. VSM-specific overexpression of *Grk5* increases blood pressure, which is sex-modified because male mice with VSM-*Grk5* overexpression have a much greater increase in blood pressure (~26 mm Hg, mean arterial pressure) than female *Grk5* mice [122]. However, the intracardiac injection of adenovirus that encodes for the RH domain within the amino terminus of *Grk5* does not reduce blood pressure levels in SHRs or in WKY rats [123]. It should be noted that the role of GRK5 in the regulation of blood pressure is complicated. Mice with global *Grk5* ablation have impaired glucose tolerance and insulin sensitivity compared with their WT littermates [124]. Therefore, GRK5 may be a positive regulator of insulin resistance, which contributes to the pathogenesis of hypertension [125]. In addition, *Grk5* overexpression augments the agonist-induced AT_1_R or D_1_R phosphorylation, leading to their desensitization; desensitization of AT_1_R should favor a lower blood pressure while the opposite is true for D_1_R and therefore, these two effects by themselves may not lead to a change in blood pressure [38, 41]. Cardiac-specific *Grk5* expression decreases basal left ventricle systolic pressure, slows left ventricle relaxation, and increases β-AR desensitization, but has no effect on Ang II-induced contractile response [32]. Thus, defining the role of GRK5 in the regulation of blood pressure needs to be studied further.

GRK5 is also associated with other blood pressure regulation-related cardiovascular diseases such as myocardial infarction or cardiometabolic traits such as hyperlipidemia [119, 126]. *Grk5* SNPs are also associated with the race-related therapeutic efficacy of some drugs, including β-adrenergic blockers [127, 128].

## GRK6

GRK6 is ubiquitously expressed in mammalian organs and tissues, including the heart and intestines [71, 129–131]. There is no direct proof of the role of GRK6 in the regulation of blood pressure. However, there are some reports on the association between GRK6 and hypertension [71]. Compared with control rats, total GRK6 expression is increased, and its distribution is reduced in the intercalated disks but increased in the cytoplasm of cardiac myocytes in spontaneously hypertensive heart failure rats [71]. This may be involved in abnormal remodeling of cardiac myocytes in hypertensive hypertrophy and failure. The cardiomyocytes of GRK6 but not GRK5 knockout mice have an augmented fractional shortening in response to Ang II [132].

Abnormal proliferation of endothelial cells is part of the vascular remodeling in hypertension that may be related to a decrease in vascular endothelial GRK6 expression caused by miR-27a [133]. Cyclic stretching of VSMs, to mimic the pathologically increased stretch in hypertension, causes the transfer of miR-27a via VSMC microparticles to the endothelial cells [133].

As aforementioned, sodium retention, caused by impaired renal sodium excretion, is part of the pathological process in hypertension [134]. An increase in intestinal sodium absorption is also part of the increased sodium balance in hypertension, which may be related to increased activity of NHE3 and Na^+^-K^+^-ATPase [135, 136]. Inhibition of GRK6, but not GRK4, prevents the loss of dopamine D_1_-like receptor-mediated inhibition of Cl/HCO3^-^ exchanger and NHE3 activity in rat intestinal epithelial cells [130, 131]. In addition, GRK6 regulates insulin homeostasis, an abnormality which participates in the pathogenesis of hypertension [137]. GRK6 inhibition increases insulin secretion but reduces insulin processing; *Grk6* knockdown reduces cellular insulin levels, and attenuates insulin secretion, but enhances proinsulin secretion consistent with decreased processing [138]. However, the proof of the association between GRK6 and hypertension is still weak and needs further exploration.

## Modulation of Grks in the regulation of blood pressure

Most studies have focused on the role of GRKs in the regulation of GPCR expression and activity. However, there are studies reporting on how GRKs are regulated. In fact, there are some studies reporting that GRKs per se are modulated by factors, which subsequently regulate blood pressure. For example, long-term exercise in SHRs, beginning at the prehypertensive stage (four weeks old), improves vascular insulin sensitivity via down-regulation of vascular GRK2, which limits the progression of hypertension [58]. Activation of the TGF-β signaling cascade in VSMCs increases GRK2 expression, consequently inhibiting Ang II-induced VSMC proliferation and migration [139]. We have reported that the expression and activity of GRKs are regulated by several factors, including long-term PM_2.5_ exposure, prenatal and paternal PM_2.5_ exposure, in utero lipopolysaccharide or cold stress exposure, and maternal diabetes mellitus [49, 87–89, 92, 94].

The expression of GRKs is regulated by transcription factors and signaling molecules. Others and we have reported that renal GRK4 expression is positively regulated by the activation of the transcription factor c-Myc, which binds to the promoter of *Grk4* gene [87, 90, 140]. Other transcription factors, such as Foxo1, NF-κB, and cAMP-responsive element binding protein, are also involved in the regulation of transcription of GRKs (GRK2, GRK5, GRK6) [141–143]. Some key signaling molecules such as protein kinases participate in the modulation of GRK expression. We reported that inhibition of PKC reduces the increased GRK2 expression and normalizes D_1_R function in renal proximal tubule cells from diabetic mother offspring, which may be beneficial in the treatment of maternal diabetes mellitus-programmed hypertension [49]. Activation of PKC increases GRK2 protein and activity, causing the dysfunction of endothelium-dependent relaxation to insulin in mouse aorta [144]. By contrast, activation of PKC decreases GRK6 promoter activity and expression [143]. Post-transcriptional regulation, including ubiquitination, also plays an important role in the regulation of GRKs [145, 146].

The regulation of GRK activity or cellular location, independent of its expression, is also important in disease states. Insulin or oxidative stress causes renal D_1_R desensitization and impairs its ability to inhibit Na^+^-K^+^-ATPase activity by promoting GRK2 translocation to the plasma membrane [48, 147]. The increased blood pressure of SHRs is caused, in part, by impaired renal D_1_R function [148], that is due to increased GRK4 activity and sorting nexins (e.g., SNX5 and SNX19) [15, 149, 150]. SNXs play a vital role in receptor trafficking [151]. SNX5 directly interacts with renal GRK4 and inhibits its ability to phosphorylate and desensitize D_1_R [149].The nuclear translocation of GRK5 participates in fibroblast activation and cardiac fibrosis; Ang II induces the nuclear translocation of cardiac GRK5 [12]. It should be noted that SNPs of GRKs also affect the activity of GRKs. *GRK4* variants can increase the activity of GRK4 [28, 77–79, 93]. As aforementioned, increased renal GRK4 activity, caused by *GRK4* gene variants, causes hypertension [15, 77, 78, 82, 83]. The *GRK4* variant *A486V* has been shown to increase the impairment of cardiac function after myocardial infarction [28]. A functional GRK2 SNP which inhibits its activity potentiates β-adrenergic receptor-mediated cAMP accumulation and potentially can be used in the treatment of heart failure [152].

## Grks and treatment of hypertension

### GRK inhibition

Because GRKs play a role in the pathogenesis of cardiovascular diseases, including hypertension, many studies have focused on searching selective inhibitors of GRKs, especially GRK2 and GRK5. Some existing drugs have been verified as potential GRK2 inhibitors. For example, paroxetine directly inhibits the activity of GRK2 by binding its active site which stabilizes the kinase domain [153]. In hypertension, paroxetine increases β_1_-AR sensitivity and attenuates cardiac hypertrophy by blocking the interaction between GRK2 and β_1_-AR [154]. The actions of some GRK inhibitors have been related to their crystal structure. For example, based on the conformation of the HJ loop within the X-ray structure of GRK2, cyclic peptide 7 was found to suppress selectively and potently GRK2 activity [155]. The pharmacological inhibition of GRK2 by a cyclic peptide C7 improves cardiac metabolism and function in experimental heart failure [156]. Small molecule GRK inhibitors, including paroxetine, Takeda compound 101, and CCG224063, attenuate GRK2-mediated desensitization of vasoconstrictor-induced arterial contractions, highlighting a potential strategy for blood pressure regulation by targeting GRK2 function [157].

Most of the current studies on the inhibition of GRK function have focused on the direct interaction between the inhibitor and the kinase domain of the protein. However, inhibitors of the RH domain of GRK2 have also been reported to reduce the RH-mediated receptor desensitization and GRK2 translocation to the plasma membrane [158, 159]. These novel inhibitors of phosphorylation-independent actions of GRK2 provide new strategies for the development of innovative pharmacologic therapy for hypertension.

Some studies have also explored the possibility of simultaneous inhibition of two types of GRKs by one inhibitor. For example, analyses of the structures and chemical properties of key residues in the protein crystal structures of GRK2 and GRK5 have determined the specific amino acid distribution within the GRK2/GRK5 target site [160, 161]. CCG215022, a GRK2 inhibitor, has nanomolar IC50 values against both GRK2 and GRK5. A 2.4 Å crystal structure of the GRK5·CCG215022 complex revealed that the inhibitor binds in the active site similarly to its parent compound [162]. The structure and function of GRK4 486V have also been reported; Ser-485 in the kinase C-tail is one of the residues that is responsible for the increased rate of autophosphorylation of GRK4 486V [84]. These are promising reports on the generation of selective GRK inhibitors in the treatment of hypertension.

### GRKs and pharmacogenomics of hypertension

Over the past decade, pharmacogenetics has been used to guide cardiovascular drug therapy [163]. The gene variants of *GRKs* may be important to guide antihypertensive strategies. The African American Study of Kidney Disease and Hypertension Study showed that in African-American men, but not women, the response to the β_1_-AR blocker metoprolol was more rapid in carriers of *GRK4 142V* than carriers of *GRK4 A142* but the response was decreased in the presence of *GRK4 65L* [111]. The Pharmacogenomic Evaluation of Antihypertensive Responses trial showed that the response of hypertensive patients to β-blockers is influenced by *GRK4* variants. In African-Americans and Caucasians, increasing number of copies of the *GRK4* variant *65L-142V* haplotype is associated with reduced response to β-blocker monotherapy with atenolol [164]. Moreover, all three *GRK4* variants (*65L, 142V, and 486V*) are associated with increased risk for the primary outcome of first occurrence of all cause death, nonfatal myocardial infarction, or nonfatal stroke in American whites and Hispanics [164]. Another study in hypertensive Japanese showed that patients with *hGRK4 142V* have a greater decrease in SBP in response to angiotensin receptor blockers (ARBs) than non-carrier subjects. However, those with *GRK4 486V* are less likely to achieve the blood pressure goal in response to ARBs than those without *GRK4* variants [165]. Our study also found that non-dipper circadian rhythm is positively associated with the presence of *GRK4* variants. Moreover, patients with these three *GRK4* variants have a better antihypertensive response to candesartan, an ARB, than those with *GRK4* WT gene [166].

Regarding the antihypertensive effect of diuretics, one study showed that subjects with more than three *GRK4* gene variants (*A142V, A486V, and R65L*) have a greater increase in potassium and sodium excretion in response to hydrochlorothiazide than those with three or less than three *GRK4* gene variants [167]. In another study, hypertensive subjects with *GRK4 65L* and *142V* needed more antihypertensive therapy, especially diuretics, to reach the same mean arterial pressure as did the heterozygous and wild-type carriers of *GRK4* [168]. Other *GRK* variants are reported to be associated with therapeutic effect of antihypertensive drugs. African-American carriers of *GRK2 rs4930416* have a greater decrease in both SBP and DBP in response to hydrochlorothiazide while European-Americans with *GRK2 rs1894111* have a greater decrease in both SBP and DBP in response to hydrochlorothiazide [16]. African-American or European-American carriers of *GRK5Gln41*, relative to homozygous carriers of *GRK5Leu41*, do not respond differently to hydrochlorothiazide or atenolol but those with *GRK5Leu41* have a 46.5% risk reduction for adverse cardiovascular outcomes compared with GRK5 *Gln41* homozygotes, independent of treatment [16].

*GRK4* variants are also associated with the blood pressure response to lifestyle modification. For example, Rayner et al. found that in South African Blacks 50-75 years old with mild-to-moderate hypertension, *GRK4* polymorphisms, *A142V and R65L*, can predict blood pressure response to dietary reduction of dietary salt intake. Hypertensive subjects with wild-type *GRK4 A142* and *GRK4 R65* or heterozygous *GRK4 A142V* and *GRK4 R65L* but no homozygous *GRK4 142V* and *GRK4 65L* have a reduction in their blood pressures in response to a low salt diet [169].

## Limitations of Grks research

There are several limitations in the current GRK research. First, there are only a few reports using organ-specific knockout or overexpression GRK mice, which are better to study the role of GRKs in the regulation of blood pressure. Second, the mechanism by which different *GRKs* polymorphisms affect enzymatic activity remains still unknown, which may be related with the crystal structure of *GRKs* polymorphisms. Especially, the reason why different GRK4 variants exhibit different salt-sensitive phenotypes in blood pressure regulation needs to be studied in the future. Third, GRK2-mediated effects in the regulation of blood pressure is complicated, and its role in hypertension needs to be clarified more clearly. Fourth, the regulatory mechanisms of GRK remain unclear.

## Conclusions

In summary, increasing pieces of evidence demonstrate that GRKs, by regulating different substrates, play important roles in the regulation of blood pressure. Various genetically modified animal models confirm the key role of GRKs in the regulation of blood pressure (Table 3). Aberrant expression and/or activity of GRKs in the cardiovascular system and kidney participate in the pathogenesis of hypertension. Genetic association studies have found associations between GRK gene variants and hypertension. Moreover, individual GRK variants also affect the response to antihypertensive treatment. Increased understanding of the role of GRKs in the regulation of blood pressure may provide novel insights into the pathogenesis of hypertension and new strategies for the prevention and treatment of hypertension.

**Table 3:** Effects of GRK modification on blood pressure and related substrates

GRK isoform	GRK Modification	Mouse Phenotype	Regulation on Substrate and Its Functions
GRK2	VSM-targeted overexpression	Increases resting SBP, DBP and MAP [34]; increases SBP but not MAP or DBP [60]	Attenuates β-AR signaling and vasodilation, increases aortic medial VSM thickness, and causes cardiac hypertrophy [34]; impairs β-AR signaling in VSM [60]
	Global adult hemizygous mice (GRK2^+/-^)	No change in basal SBP, but reduced Ang II-induced hypertension	Increases aortic vasoconstrictor responses to phenylephrine and ET-1 but not to Ang II; increases endothelium-dependent vasodilator responses and NO release; prevents Ang II-induced vascular remodeling [31]
	Global knockdown *Grk2* using shRNA	Causes hypertension [33, 43]	Increases phenylephrine-induced hypertension and contractile response of mesenteric arteries and isoproterenol-mediated vasodilation in phenylephrine pre-constricted mesenteric arteries; decreases desensitization of β-AR signaling in VSMCs [33]; decreases kidney size, nephrogenesis and glomerular count, and glomerular filtration; increases RAS activity and renin- and AT_1_R-mediated ROS production; does not affect renal AT_1_R expression [43]
	GRK2ct or VSM-selective *Grk2* ablation	no effect on basal blood pressure; does not prevent 2K1C hypertension [39]	Increases β-AR mediated dilation and α_1D_-AR stimulated vasoconstriction [39]
	Selective deletion of endothelial *Grk2*	not mentioned	Impairs α_1_-AR-, serotonin-, oxytocin-, and KCl-induced aortic vasoconstriction; increases aortic inflammation, degeneration, and mitochondrial ROS production [59]
	*Grk2* knockdown using siRNA	not mentioned	Improves insulin-induced vasodilation of mesenteric arteries in SHRs [58]
GRK3	Cardiac-restricted expression of a peptide inhibitor of *GRK3* in mice	Increases SBP, DBP, and cardiac output	Does not affect cardiac β-AR responsiveness; increases α_1_-AR but not β-AR -induced cardiac hypertrophy [73]; attenuates cardiac dysfunction and heart failure after chronic pressure overload [74]
GRK4	Global *Grk4* knockout	Decreases SBP and DBP	Cardiac and renal function not studied [78]
	Renal depletion using As-Odns	Attenuates SBP and MAP in SHRs [79]	Decreases renal D_1_R phosphorylation, increases sodium excretion and urine volume, and reduces protein excretion in SHRs [79]
	UTMD-targeted renal depletion of *Grk* in rodents	Reduces SBP [81]	Reduces the phosphorylation of some receptors expressed in the kidney (D_1_R, adipoR1, ETBR, AT_2_R) and improves receptor-mediated natriuresis and diuresis [21, 81, 82, 93]
	Overexpression of human *GRK4γ 142V* in mice	Causes hypertension (normal-salt diet); increases systolic blood pressure in response to Ang II [77]	Impairs renal receptor (D_1_R, adipoR1, ETBR, AT_2_R and CCKBR)-mediated sodium excretion [15, 21, 81, 82, 93, 170]; increases arterial AT_1_R expression and -mediated vasoconstriction [77]; increases renal AT_1_R expression and -induced sodium retention [78]
	Overexpression of human *GRK4 γ 486V* in mice	Salt-sensitive hypertension [83]	Impairs sodium excretion and increases renal oxidative stress [83]; increases renal AT_1_R expression and activity [83]
	Overexpression of human *GRK4 65L* in mice	Salt-sensitive hypertension	Increases D_1_R phosphorylation [174], increases renal Na^+^,K^+^/ATPase and α-ENaC expressions [175]
GRK5	VSM-specific Overexpression in mice	Causes hypertension; increases MAP to a greater extent in male than female mice	Increases norepinephrine-mediated vasoconstriction in male mice; Decreases βAR-mediated vasodilation and increases Ang II–stimulated vasoconstriction in female but not male mice [122]
	AdGRK5-NT in rats	Does not affect the blood pressures of WKY and SHRs [123]	Decreases cardiac mass in SHRs and prevents the development of phenylephrine-induced left cardiac hypertrophy, inhibits NF-κB signaling, transcriptional activity and cardiac fibrosis and apoptosis [123]
	Global knockout mice	Not mentioned	Decreases insulin sensitivity [124]
GRK6	Global knockout mice	Not mentioned	Causes striatal D_2_-like receptor supersensitivity [176] Increases ear inflammation [177]

*α-AR* α-adrenergic receptor, *AdGRK5-NT* an adenovirus that encodes for the RH domain within the amino terminal of GRK5, adipoR1 adiponectin receptor 1, *α-ENaC* α-epithelial sodium channel, *Ang II* angiotensin II, *As-Odns* antisense oligodeoxynucleotides, *AT*_*1*_*R* angiotensin II type 1 receptor, *AT*_*2*_*R* angiotensin II type 2 receptor, *β-AR* β-adrenergic receptor, *ct* carboxyterminal, *CCKBR* cholecystokinin receptor type B, *D*_*1*_*R* dopamine D_1_ receptor, *DBP* diastolic blood pressure, *ET-1* endothelin-1, *ETBR* endothelin receptor type B, *GRK* G protein-coupled receptor kinase, *GRK2ct* a peptide inhibitor composed of the carboxyl-terminal portion of GRK2, *2K1C* two-kidney one-clip, *KCl* potassium chloride, *MAP* mean arterial blood pressure, *NF-κB* nuclear factor-κB, *NO* nitric oxide, *RAS* renin-angiotensin system, *ROS* reactive oxygen species, *SBP* systolic blood pressure, *ShRNA* small hairpin RNA, *SHRs* spontaneously hypertensive rats, *siRNA* small interfering RNA, *VSM* vascular smooth muscle, *VSMCs* vascular smooth muscle cells, *UTMD* ultrasound-targeted microbubble destruction

## References

[1] Brouwers S, Sudano I, Kokubo Y, Sulaica EM. Arterial hypertension. Lancet. 2021;398:249–61. 34019821 10.1016/S0140-6736(21)00221-X

[2] NCD Risk Factor Collaboration (NCD-RisC). Worldwide trends in hypertension prevalence and progress in treatment and control from 1990 to 2019: a pooled analysis of 1201 population-representative studies with 104 million participants. Lancet. 2021;398:957–80. 34450083 10.1016/S0140-6736(21)01330-1 PMC8446938

[3] Tsao CW, Aday AW, Almarzooq ZI, Anderson CAM, Arora P, Avery CL, et al. Heart disease and stroke statistics-2023 update: a report from the American Heart Association. Circulation. 2023;147:e93–e621. 36695182 10.1161/CIR.0000000000001123 PMC12135016

[4] Carey RM, Moran AE, Whelton PK. Treatment of hypertension: a review. JAMA. 2022;328:1849–61. 36346411 10.1001/jama.2022.19590

[5] Grogan A, Lucero EY, Jiang H, Rockman HA. Pathophysiology and pharmacology of G protein-coupled receptors in the heart. Cardiovasc Res. 2023;119:1117–29. 36534965 10.1093/cvr/cvac171 PMC10202650

[6] Jiang H, Galtes D, Wang J, Rockman HA. G protein-coupled receptor signaling: transducers and effectors. Am J Physiol Cell Physiol. 2022;323:C731–C748. 35816644 10.1152/ajpcell.00210.2022 PMC9448338

[7] Vaz de Castro PAS, Jose PA, Simões E Silva AC. Interactions between the intrarenal dopaminergic and the renin-angiotensin systems in the control of systemic arterial pressure. Clin Sci (Lond). 2022;136:1205–27. 35979889 10.1042/CS20220338

[8] Yang J, Villar VAM, Jose PA, Zeng C. Renal dopamine receptors and oxidative stress: role in hypertension. Antioxid Redox Signal. 2021;34:716–35. 32349533 10.1089/ars.2020.8106 PMC7910420

[9] Banday AA, Lokhandwala MF. Transcriptional regulation of renal dopamine D_1_ receptor function during oxidative stress. Hypertension. 2015;65:1064–72. 25733244 10.1161/HYPERTENSIONAHA.115.05255 PMC4393374

[10] Kemp BA, Howell NL, Gildea JJ, Keller SR, Brautigan DL, Carey RM, et al. _2_ receptors mediate natriuresis via protein phosphatase PP2A. Circ Res. 2022;130:96–111. 34794320 10.1161/CIRCRESAHA.121.319519 PMC8741733

[11] Pfleger J, Gresham K, Koch WJ. G protein-coupled receptor kinases as therapeutic targets in the heart. Nat Rev Cardiol. 2019;16:612–22. 31186538 10.1038/s41569-019-0220-3

[12] Eguchi A, Coleman R, Gresham K, Gao E, Ibetti J, Chuprun JK, et al. GRK5 is a regulator of fibroblast activation and cardiac fibrosis. Proc Natl Acad Sci USA. 2021;118:e2012854118. 33500351 10.1073/pnas.2012854118 PMC7865138

[13] Yang J, Hall JE, Jose PA, Chen K, Zeng C. Comprehensive insights in GRK4 and hypertension: from mechanisms to potential therapeutics. Pharm Ther. 2022;239:108194. 10.1016/j.pharmthera.2022.108194 PMC9728143 35487286

[14] Gros R, Benovic JL, Tan CM, Feldman RD. G-protein-coupled receptor kinase activity is increased in hypertension. J Clin Investig. 1997;99:2087–93. 9151780 10.1172/JCI119381 PMC508038

[15] Felder RA, Sanada H, Xu J, Yu PY, Wang Z, Watanabe H, et al. G protein-coupled receptor kinase 4 gene variants in human essential hypertension. Proc Natl Acad Sci USA. 2002;99:3872–7. 11904438 10.1073/pnas.062694599 PMC122616

[16] Lobmeyer MT, Wang L, Zineh I, Turner ST, Gums JG, Chapman AB, et al. Polymorphisms in genes coding for GRK2 and GRK5 and response differences in antihypertensive-treated patients. Pharmacogenet Genom. 2011;21:42–49. 10.1097/FPC.0b013e328341e911 PMC3028503 21127457

[17] Yang J, Villar VA, Armando I, Jose PA, Zeng C. G protein-coupled receptor kinases: crucial regulators of blood pressure. J Am Heart Assoc. 2016;5:e003519. 27390269 10.1161/JAHA.116.003519 PMC5015388

[18] Sulon SM, Benovic JL, Targeting G. protein-coupled receptor kinases (GRKs) to G protein-coupled receptors. Curr Opin Endocr Metab Res. 2021;16:56–65. 33718657 10.1016/j.coemr.2020.09.002 PMC7945687

[19] Benovic JL. Historical perspective of the G protein-coupled receptor kinase family. Cells. 2021;10:555. 33806476 10.3390/cells10030555 PMC7999923

[20] Chaudhary PK, Kim S. The GRKs reactome: role in cell biology and pathology. Int J Mol Sci. 2021;22:3375. 33806057 10.3390/ijms22073375 PMC8036551

[21] Zhang Y, Wang S, Huang H, Zeng A, Han Y, Zeng C, et al. GRK4-mediated adiponectin receptor-1 phosphorylative desensitization as a novel mechanism of reduced renal sodium excretion in hypertension. Clin Sci. 2020;134:2453–67. 10.1042/CS20200671 PMC7654732 32940654

[22] Crudden C, Shibano T, Song D, Dragomir MP, Cismas S, Serly J, et al. Inhibition of G protein-coupled receptor kinase 2 promotes unbiased downregulation of IGF1 receptor and restrains malignant cell growth. Cancer Res. 2021;81:501–14. 33158816 10.1158/0008-5472.CAN-20-1662

[23] Usui I, Imamura T, Babendure JL, Satoh H, Lu JC, Hupfeld CJ, et al. G protein-coupled receptor kinase 2 mediates endothelin-1-induced insulin resistance via the inhibition of both G alpha q/11 and insulin receptor substrate-1 pathways in 3T3-L1 adipocytes. Mol Endocrinol. 2005;19:2760–8. 15994203 10.1210/me.2004-0429

[24] Martini JS, Raake P, Vinge LE, DeGeorge BR Jr, Chuprun JK, Harris DM, et al. Uncovering G protein-coupled receptor kinase-5 as a histone deacetylase kinase in the nucleus of cardiomyocytes. Proc Natl Acad Sci USA. 2008;105:12457–62. 18711143 10.1073/pnas.0803153105 PMC2527933

[25] Patial S, Luo J, Porter KJ, Benovic JL, Parameswaran N. G-protein-coupled-receptor kinases mediate TNFα-induced NFκB signalling via direct interaction with and phosphorylation of IκBα. Biochem J. 2009;425:169–78. 19796012 10.1042/BJ20090908 PMC2856098

[26] Gurevich EV, Tesmer JJ, Mushegian A, Gurevich VV. G protein-coupled receptor kinases: more than just kinases and not only for GPCRs. Pharm Ther. 2012;133:40–69. 10.1016/j.pharmthera.2011.08.001 PMC3241883 21903131

[27] Yang D, Tang M, Zhang M, Ren H, Li X, Zhang Z, et al. Downregulation of G protein-coupled receptor kinase 4 protects against kidney ischemia-reperfusion injury. Kidney Int. 2023;103:719–34. 36669643 10.1016/j.kint.2022.12.023 PMC12621542

[28] Li L, Fu W, Gong X, Chen Z, Tang L, Yang D, et al. The role of G protein-coupled receptor kinase 4 in cardiomyocyte injury after myocardial infarction. Eur Heart J. 2021;42:1415–30. 33280021 10.1093/eurheartj/ehaa878 PMC8026279

[29] Gerhards J, Maerz LD, Matthees ESF, Donow C, Moepps B, Premont RT, et al. Kinase activity is not required for G protein-coupled receptor kinase 4 restraining mTOR signaling during cilia and kidney development. J Am Soc Nephrol. 2023;34:590–606. 36810260 10.1681/ASN.0000000000000082 PMC10103308

[30] Yang J, Villar VA, Jones JE, Jose PA, Zeng C. G protein-coupled receptor kinase 4: role in hypertension. Hypertension. 2015;65:1148–55. 25870190 10.1161/HYPERTENSIONAHA.115.05189 PMC6350509

[31] Avendaño MS, Lucas E, Jurado-Pueyo M, Martínez-Revelles S, Vila-Bedmar R, Mayor F Jr, et al. Increased nitric oxide bioavailability in adult GRK2 hemizygous mice protects against angiotensin II-induced hypertension. Hypertension. 2014;63:369–75. 24191280 10.1161/HYPERTENSIONAHA.113.01991

[32] Rockman HA, Choi DJ, Rahman NU, Akhter SA, Lefkowitz RJ, Koch WJ. Receptor-specific in vivo desensitization by the G protein-coupled receptor kinase-5 in transgenic mice. Proc Natl Acad Sci USA. 1996;93:9954–9. 8790438 10.1073/pnas.93.18.9954 PMC38536

[33] Tutunea-Fatan E, Caetano FA, Gros R, Ferguson SSG. GRK2 targeted knock-down results in spontaneous hypertension, and altered vascular GPCR signaling. J Biol Chem. 2015;290:5141–55. 25561731 10.1074/jbc.M114.615658 PMC4335248

[34] Eckhart AD, Ozaki T, Tevaearai H, Rockman HA, Koch WJ. Vascular-targeted overexpression of G protein-coupled receptor kinase-2 in transgenic mice attenuates beta-adrenergic receptor signaling and increases resting blood pressure. Mol Pharm. 2002;61:749–58. 10.1124/mol.61.4.749 11901213

[35] Morris GE, Nelson CP, Standen NB, Challiss RA, Willets JM. Endothelin signalling in arterial smooth muscle is tightly regulated by G protein-coupled receptor kinase 2. Cardiovasc Res. 2010;85:424–33. 19748906 10.1093/cvr/cvp310 PMC2802200

[36] Morris GE, Nelson CP, Everitt D, Brighton PJ, Standen NB, Challiss RA, et al. G protein-coupled receptor kinase 2 and arrestin2 regulate arterial smooth muscle P2Y-purinoceptor signalling. Cardiovasc Res. 2011;89:193–203. 20705669 10.1093/cvr/cvq249 PMC3002865

[37] Morris GE, Nelson CP, Brighton PJ, Standen NB, Challiss RA, Willets JM. Arrestins 2 and 3 differentially regulate ETA and P2Y2 receptor-mediated cell signaling and migration in arterial smooth muscle. Am J Physiol Cell Physiol. 2012;302:C723–C734. 22159081 10.1152/ajpcell.00202.2011 PMC3311302

[38] Oppermann M, Freedman NJ, Alexander RW, Lefkowitz RJ. Phosphorylation of the type 1A angiotensin II receptor by G protein-coupled receptor kinases and protein kinase C. J Biol Chem. 1996;271:13266–72. 8662816 10.1074/jbc.271.22.13266

[39] Cohn HI, Harris DM, Pesant S, Pfeiffer M, Zhou RH, Koch WJ, et al. Inhibition of vascular smooth muscle G protein-coupled receptor kinase 2 enhances alpha1D-adrenergic receptor constriction. Am J Physiol Heart Circ Physiol. 2008;295:H1695–H1704. 18723764 10.1152/ajpheart.00564.2008 PMC2593515

[40] Nash CA, Nelson CP, Mistry R, Moeller-Olsen C, Christofidou E, Challiss RAJ, et al. Differential regulation of β_2_-adrenoceptor and adenosine A2B receptor signalling by GRK and arrestin proteins in arterial smooth muscle. Cell Signal. 2018;51:86–98. 30075183 10.1016/j.cellsig.2018.07.013

[41] Tiberi M, Nash SR, Bertrand L, Lefkowitz RJ, Caron MG. Differential regulation of dopamine D1A receptor responsiveness by various G protein-coupled receptor kinases. J Biol Chem. 1996;271:3771–8. 8631993 10.1074/jbc.271.7.3771

[42] Taguchi K, Bessho N, Hasegawa M, Narimatsu H, Matsumoto T, Kobayashi T. Co-treatment with clonidine and a GRK2 inhibitor prevented rebound hypertension and endothelial dysfunction after withdrawal in diabetes. Hypertens Res. 2018;41:263–74. 29463871 10.1038/s41440-018-0016-6

[43] Tutunea-Fatan E, Abd-Elrahman KS, Thibodeau JF, Holterman CE, Holleran BJ, Leduc R, et al. GRK2 knockdown in mice exacerbates kidney injury and alters renal mechanisms of blood pressure regulation. Sci Rep. 2018;8:11415. 30061705 10.1038/s41598-018-29876-8 PMC6065385

[44] Dinudom A, Fotia AB, Lefkowitz RJ, Young JA, Kumar S, Cook DI. The kinase Grk2 regulates Nedd4/Nedd4-2-dependent control of epithelial Na^+^ channels. Proc Natl Acad Sci USA. 2004;101:11886–90. 15284439 10.1073/pnas.0402178101 PMC511069

[45] Pitzer AL, Van Beusecum JP, Kleyman TR, Kirabo A. ENaC in salt-sensitive hypertension: kidney and beyond. Curr Hypertens Rep. 2020;22:69. 32852643 10.1007/s11906-020-01067-9 PMC7452925

[46] Sanchez-Perez A, Kumar S, Cook DI. GRK2 interacts with and phosphorylates Nedd4 and Nedd4-2. Biochem Biophys Res Commun. 2007;359:611–5. 17544362 10.1016/j.bbrc.2007.05.134

[47] Asghar M, Banday AA, Fardoun RZ, Lokhandwala MF. Hydrogen peroxide causes uncoupling of dopamine D_1_-like receptors from G proteins via a mechanism involving protein kinase C and G-protein-coupled receptor kinase 2. Free Radic Biol Med. 2006;40:13–20. 16337875 10.1016/j.freeradbiomed.2005.08.018

[48] Banday AA, Fazili FR, Lokhandwala MF. Insulin causes renal dopamine D_1_ receptor desensitization via GRK2-mediated receptor phosphorylation involving phosphatidylinositol 3-kinase and protein kinase C. Am J Physiol Ren Physiol. 2007;293:F877–F884. 10.1152/ajprenal.00184.2007 17567939

[49] Luo H, Chen C, Guo L, Xu Z, Peng X, Wang X, et al. Exposure to maternal diabetes mellitus causes renal dopamine D_1_ receptor dysfunction and hypertension in adult rat offspring. Hypertension. 2018;72:962–70. 30354705 10.1161/HYPERTENSIONAHA.118.10908 PMC6207228

[50] Zhu W, Petrashevskaya N, Ren S, Zhao A, Chakir K, Gao E, et al. Gi-biased β_2_AR signaling links GRK2 upregulation to heart failure. Circ Res. 2012;110:265–74. 22179058 10.1161/CIRCRESAHA.111.253260 PMC3282829

[51] Guo S, Carter RL, Grisanti LA, Koch WJ, Tilley DG. Impact of paroxetine on proximal β-adrenergic receptor signaling. Cell Signal. 2017;38:127–33. 28711716 10.1016/j.cellsig.2017.07.006 PMC5646168

[52] Wang Y, Gao E, Lau WB, Wang Y, Liu G, Li JJ, et al. G-protein-coupled receptor kinase 2-mediated desensitization of adiponectin receptor 1 in failing heart. Circulation. 2015;131:1392–404. Apr 21 25696921 10.1161/CIRCULATIONAHA.114.015248 PMC4406820

[53] Chen X, Zhao S, Xia Y, Xiong Z, Li Y, Tao L, et al. G protein coupled receptor kinase-2 upregulation causes κ-opioid receptor desensitization in diabetic heart. Biochem Biophys Res Commun. 2017;482:658–64. 27865836 10.1016/j.bbrc.2016.11.090

[54] Pfleger J, Gross P, Johnson J, Carter RL, Gao E, Tilley DG, et al. G protein-coupled receptor kinase 2 contributes to impaired fatty acid metabolism in the failing heart. J Mol Cell Cardiol. 2018;123:108–17. 30171848 10.1016/j.yjmcc.2018.08.025

[55] Liu J, Li X, Ding L, Li W, Niu X, Gao D. GRK2 participation in cardiac hypertrophy induced by isoproterenol through the regulation of Nrf2 signaling and the promotion of NLRP3 inflammasome and oxidative stress. Int Immunopharmacol. 2023;117:109957. 37012864 10.1016/j.intimp.2023.109957

[56] Bledzka KM, Manaserh IH, Grondolsky J, Pfleger J, Roy R, Gao E, et al. A peptide of the amino-terminus of GRK2 induces hypertrophy and yet elicits cardioprotection after pressure overload. J Mol Cell Cardiol. 2021;154:137–53. 33548241 10.1016/j.yjmcc.2021.01.004 PMC8101069

[57] Alonazi ASA, Willets JM. G protein-coupled receptor kinase 2 is essential to enable vasoconstrictor-mediated arterial smooth muscle proliferation. Cell Signal. 2021;88:110152. 34555505 10.1016/j.cellsig.2021.110152

[58] Xing W, Li Y, Zhang H, Mi C, Hou Z, Quon MJ, et al. Improvement of vascular insulin sensitivity by downregulation of GRK2 mediates exercise-induced alleviation of hypertension in spontaneously hypertensive rats. Am J Physiol Heart Circ Physiol. 2013;305:H1111–H1119. 23913704 10.1152/ajpheart.00290.2013 PMC4747915

[59] Ciccarelli M, Sorriento D, Franco A, Fusco A, Del Giudice C, Annunziata R, et al. Endothelial G protein-coupled receptor kinase 2 regulates vascular homeostasis through the control of free radical oxygen species. Arterioscler Thromb Vasc Biol. 2013;33:2415–24. 23950144 10.1161/ATVBAHA.113.302262 PMC4262246

[60] Yano H, Onoue K, Tokinaga S, Ioka T, Ishihara S, Hashimoto Y, et al. Overexpression of GRK2 in vascular smooth muscle leads to inappropriate hypertension and acute heart failure as in clinical scenario 1. Sci Rep. 2023;13:7707. 37173348 10.1038/s41598-023-34209-5 PMC10182096

[61] Zhao Y, Vanhoutte PM, Leung SW. α_1_-Adrenoceptor activation of PKC-ε causes heterologous desensitization of thromboxane receptors in the aorta of spontaneously hypertensive rats. Br J Pharm. 2015;172:3687–701. 10.1111/bph.13157 PMC4507169 25857252

[62] Montó F, Oliver E, Vicente D, Buendía F, Rueda J, Agüero J, et al. β_2_- and β_1_-adrenoceptor expression exhibits a common regulatory pattern with GRK2 and GRK5 in human and animal models of cardiovascular diseases. J Cardiovasc Pharm. 2015;66:478–86. 10.1097/FJC.0000000000000299 26248277

[63] Gros R, Chorazyczewski J, Meek MD, Benovic JL, Ferguson SS, Feldman RD. G-Protein-coupled receptor kinase activity in hypertension : increased vascular and lymphocyte G-protein receptor kinase-2 protein expression. Hypertension. 2000;35:38–42. 10642272 10.1161/01.HYP.35.1.38

[64] Oliver E, Flacco N, Arce C, Ivorra MD, D’Ocon MP, Noguera MA. Changes in adrenoceptors and G-protein-coupled receptor kinase 2 in L-NAME-induced hypertension compared to spontaneous hypertension in rats. J Vasc Res. 2014;51:209–20. 24942010 10.1159/000360400

[65] Gros R, Tan CM, Chorazyczewski J, Kelvin DJ, Benovic JL, Feldman RD. G-protein-coupled receptor kinase expression in hypertension. Clin Pharm Ther. 1999;65:545–51. 10.1016/S0009-9236(99)70074-3 10340920

[66] Izzo R, Cipolletta E, Ciccarelli M, Campanile A, Santulli G, Palumbo G, et al. Enhanced GRK2 expression and desensitization of beta AR vasodilatation in hypertensive patients. Clin Transl Sci. 2008;1:215–20. 20443852 10.1111/j.1752-8062.2008.00050.x PMC5350663

[67] Cohn HI, Xi Y, Pesant S, Harris DM, Hyslop T, Falkner B, et al. G protein-coupled receptor kinase 2 expression and activity are associated with blood pressure in Black Americans. Hypertension. 2009;54:71–76. 19487588 10.1161/HYPERTENSIONAHA.108.125955 PMC2745090

[68] Li Y, Li N, Yao X, Heizati M, Zhang D, Zhu Q, et al. Association between polymorphisms of ADRBK1 gene and plasma renin activity in hypertensive patients: a case-control study. Med Sci Monit. 2016;22:2981–8. 27555048 10.12659/MSM.896579 PMC5008737

[69] Napolitano R, Campanile A, Sarno L, Anastasio A, Maruotti GM, Morlando M, et al. GRK2 levels in umbilical arteries of pregnancies complicated by gestational hypertension and preeclampsia. Am J Hypertens. 2012;25:366–71. 22089113 10.1038/ajh.2011.211

[70] Lv Z, Xiong LL, Qin X, Zhang H, Luo X, Peng W, et al. Role of GRK2 in trophoblast necroptosis and spiral artery remodeling: implications for preeclampsia pathogenesis. Front Cell Dev Biol. 2021;9:694261. 34917606 10.3389/fcell.2021.694261 PMC8670385

[71] Yi XP, Zhou J, Baker J, Wang X, Gerdes AM, Li F. Myocardial expression and redistribution of GRKs in hypertensive hypertrophy and failure. Anat Rec A Discov Mol Cell Evol Biol. 2005;282:13–23. 15584034 10.1002/ar.a.20143

[72] Oliver E, Rovira E, Montó F, Valldecabres C, Julve R, Muedra V, et al. beta-Adrenoceptor and GRK3 expression in human lymphocytes is related to blood pressure and urinary albumin excretion. J Hypertens. 2010;28:1281–9. 20216086 10.1097/HJH.0b013e3283383564

[73] Vinge LE, von Lueder TG, Aasum E, Qvigstad E, Gravning JA, How OJ, et al. Cardiac-restricted expression of the carboxyl-terminal fragment of GRK3 uncovers distinct functions of GRK3 in regulation of cardiac contractility and growth: GRK3 controls cardiac alpha1-adrenergic receptor responsiveness. J Biol Chem. 2008;283:10601–10. 18165681 10.1074/jbc.M708912200

[74] von Lueder TG, Gravning J, How OJ, Vinge LE, Ahmed MS, Krobert KA, et al. Cardiomyocyte-restricted inhibition of G protein-coupled receptor kinase-3 attenuates cardiac dysfunction after chronic pressure overload. Am J Physiol Heart Circ Physiol. 2012;303:H66–H74. 22542621 10.1152/ajpheart.00724.2011

[75] Sedaghat K, Tiberi M. Cytoplasmic tail of D_1_ dopaminergic receptor differentially regulates desensitization and phosphorylation by G protein-coupled receptor kinase 2 and 3. Cell Signal. 2011;23:180–92. 20837135 10.1016/j.cellsig.2010.09.002

[76] Maimari T, Krasel C, Bünemann M, Lorenz K. The N-termini of GRK2 and GRK3 simulate the stimulating effects of RKIP on β-adrenoceptors. Biochem Biophys Res Commun. 2019;520:327–32. 31604529 10.1016/j.bbrc.2019.09.135

[77] Chen K, Fu C, Chen C, Liu L, Ren H, Han Y, et al. Role of GRK4 in the regulation of arterial AT_1_ receptor in hypertension. Hypertension. 2014;63:289–96. 24218433 10.1161/HYPERTENSIONAHA.113.01766 PMC3932160

[78] Wang Z, Zeng C, Villar VA, Chen SY, Konkalmatt P, Wang X, et al. Human GRK4γ142V variant promotes AT_1_R-mediated hypertension via renal HDAC1 inhibition. Hypertension. 2016;67:325–34. 26667412 10.1161/HYPERTENSIONAHA.115.05962 PMC4713262

[79] Sanada H, Yatabe J, Midorikawa S, Katoh T, Hashimoto S, Watanabe T, et al. Amelioration of genetic hypertension by suppression of renal G protein-coupled receptor kinase type 4 expression. Hypertension. 2006;47:1131–9. 16636192 10.1161/01.HYP.0000222004.74872.17

[80] Yatabe J, Sanada H, Midorikawa S, Hashimoto S, Watanabe T, Andrews PM, et al. Effects of decreased renal cortical expression of G protein-coupled receptor kinase 4 and angiotensin type 1 receptors in rats. Hypertens Res. 2008;31:1455–64. 18957817 10.1291/hypres.31.1455 PMC3731072

[81] Huang H, Li X, Zheng S, Chen Y, Chen C, Wang J, et al. Downregulation of renal G protein-coupled receptor kinase type 4 expression via ultrasound-targeted microbubble destruction lowers blood pressure in spontaneously hypertensive rats. J Am Heart Assoc. 2016;5:e004028. 27792639 10.1161/JAHA.116.004028 PMC5121504

[82] Yang Y, Li M, Zou X, Chen C, Zheng S, Fu C, et al. Role of GRK4 in the regulation of the renal ETB receptor in hypertension. FASEB J. 2020;34:11594–604. 32687659 10.1096/fj.201902552R PMC7725963

[83] Diao Z, Asico LD, Villar VAM, Zheng X, Cuevas S, Armando I, et al. Increased renal oxidative stress in salt-sensitive human GRK4γ486V transgenic mice. Free Radic Biol Med. 2017;106:80–90. 28189851 10.1016/j.freeradbiomed.2017.02.021 PMC5376361

[84] Allen SJ, Parthasarathy G, Darke PL, Diehl RE, Ford RE, Hall DL, et al. Structure and function of the hypertension variant A486V of G protein-coupled receptor kinase 4. J Biol Chem. 2015;290:20360–73. 26134571 10.1074/jbc.M115.648907 PMC4536442

[85] Rankin ML, Marinec PS, Cabrera DM, Wang Z, Jose PA, Sibley DR. The D_1_ dopamine receptor is constitutively phosphorylated by G protein-coupled receptor kinase 4. Mol Pharm. 2006;69:759–69. 10.1124/mol.105.019901 16338988

[86] Watanabe H, Xu J, Bengra C, Jose PA, Felder RA. Desensitization of human renal D_1_ dopamine receptors by G protein-coupled receptor kinase 4. Kidney Int. 2002;62:790–8. 12164861 10.1046/j.1523-1755.2002.00525.x

[87] Ye Z, Lu X, Deng Y, Wang X, Zheng S, Ren H, et al. In Utero exposure to fine particulate matter causes hypertension due to impaired renal dopamine D_1_ receptor in offspring. Cell Physiol Biochem. 2018;46:148–59. 29614490 10.1159/000488418 PMC6437669

[88] Wang X, Luo H, Chen C, Chen K, Wang J, Cai Y, et al. Prenatal lipopolysaccharide exposure results in dysfunction of the renal dopamine D_1_ receptor in offspring. Free Radic Biol Med. 2014;76:242–50. 25236748 10.1016/j.freeradbiomed.2014.08.010 PMC6873924

[89] Sun D, Chen K, Wang J, Zhou L, Zeng C. In-utero cold stress causes elevation of blood pressure via impaired vascular dopamine D_1_ receptor in offspring. Clin Exp Hypertens. 2020;42:99–104. 30698033 10.1080/10641963.2019.1571603

[90] Tao Y, Luo W, Chen Y, Chen C, Chen S, Li X, et al. Exercise ameliorates skeletal muscle insulin resistance by modulating GRK4-mediated D_1_R expression. Clin Sci. 2023;137:1391–407. 10.1042/CS20230664 37622333

[91] Villar VA, Jones JE, Armando I, Palmes-Saloma C, Yu P, Pascua AM, et al. G protein-coupled receptor kinase 4 (GRK4) regulates the phosphorylation and function of the dopamine D_3_ receptor. J Biol Chem. 2009;284:21425–34. 19520868 10.1074/jbc.M109.003665 PMC2755867

[92] Hu C, Tao Y, Deng Y, Cai Q, Ren H, Yu C, et al. Paternal long-term PM_2.5_ exposure causes hypertension via increased renal AT_1_R expression and function in male offspring. Clin Sci (Lond). 2021;135:2575–88. 34779863 10.1042/CS20210802 PMC8628185

[93] Zhang F, Lei L, Huang J, Wang W, Su Q, Yan H, et al. G-protein-coupled receptor kinase 4 causes renal angiotensin II type 2 receptor dysfunction by increasing its phosphorylation. Clin Sci. 2022;136:989–1003. 10.1042/CS20220236 PMC9793447 35695067

[94] Lu X, Ye Z, Zheng S, Ren H, Zeng J, Wang X, et al. Long-term exposure of fine particulate matter causes hypertension by impaired renal D_1_ receptor-mediated sodium excretion via upregulation of G-protein-coupled receptor kinase type 4 expression in Sprague-Dawley rats. J Am Heart Assoc. 2018;7:e007185. 29307864 10.1161/JAHA.117.007185 PMC5778966

[95] Chugh G, Lokhandwala MF, Asghar M. Altered functioning of both renal dopamine D_1_ and angiotensin II type 1 receptors causes hypertension in old rats. Hypertension. 2012;59:1029–36. 22411927 10.1161/HYPERTENSIONAHA.112.192302 PMC3331925

[96] Escano CS, Armando I, Wang X, Asico LD, Pascua A, Yang Y, et al. Renal dopaminergic defect in C57Bl/6J mice. Am J Physiol Regul Integr Comp Physiol. 2009;297:R1660–R1669. 19726707 10.1152/ajpregu.00147.2009 PMC2803619

[97] Jones ES, Spence JD, Mcintyre AD, Nondi J, Gogo K, Akintunde A, et al. High frequency of variants of candidate genes in Black Africans with low renin-resistant hypertension. Am J Hypertens. 2017;30:478–83. 28052878 10.1093/ajh/hpw167

[98] Kimura L, Angeli CB, Auricchio MT, Fernandes GR, Pereira AC, Vicente JP, et al. Multilocus family-based association analysis of seven candidate polymorphisms with essential hypertension in an African-derived semi-isolated Brazilian population. Int J Hypertens. 2012;2012:859219. 23056922 10.1155/2012/859219 PMC3463917

[99] Sanada H, Yatabe J, Midorikawa S, Hashimoto S, Watanabe T, Moore JH, et al. Single-nucleotide polymorphisms for diagnosis of salt-sensitive hypertension. Clin Chem. 2006;52:352–60. 16439609 10.1373/clinchem.2005.059139

[100] Gu D, Su S, Ge D, Chen S, Huang J, Li B, et al. Association study with 33 single-nucleotide polymorphisms in 11 candidate genes for hypertension in Chinese. Hypertension. 2006;47:1147–54. 16636198 10.1161/01.HYP.0000219041.66702.45

[101] Speirs HJ, Katyk K, Kumar NN, Benjafield AV, Wang WY, Morris BJ. Association of G-protein-coupled receptor kinase 4 haplotypes, but not HSD3B1 or PTP1B polymorphisms, with essential hypertension. J Hypertens. 2004;22:931–6. 15097232 10.1097/00004872-200405000-00014

[102] Bengra C, Mifflin TE, Khripin Y, Manunta P, Williams SM, Jose PA, et al. Genotyping of essential hypertension single-nucleotide polymorphisms by a homogeneous PCR method with universal energy transfer primers. Clin Chem. 2002;48:2131–40. 12446468 10.1093/clinchem/48.12.2131

[103] Jeong S, Kim JY, Cho Y, Koh SB, Kim N, Choi JR. Genetically, dietary sodium intake is causally associated with salt-sensitive hypertension risk in a community-based cohort study: a mendelian randomization approach. Curr Hypertens Rep. 2020;22:45. 32591971 10.1007/s11906-020-01050-4

[104] Jiang W, Wang X, Li R, Wang P, Shan G, Jia X, et al. Targeted capture sequencing identifies genetic variations of GRK4 and RDH8 in Han Chinese with essential hypertension in Xinjiang. PLoS One. 2021;16:e0255311. 34297769 10.1371/journal.pone.0255311 PMC8301621

[105] Du B, Jia X, Tian W, Yan X, Wang N, Cai D, et al. Associations of SUCNR1, GRK4, CAMK1D gene polymorphisms and the susceptibility of type 2 diabetes mellitus and essential hypertension in a northern Chinese Han population. J Diabetes Complic. 2021;35:107752. 10.1016/j.jdiacomp.2020.107752 33127268

[106] Zhang H, Sun ZQ, Liu SS, Yang LN. Association between GRK4 and DRD1 gene polymorphisms and hypertension: a meta-analysis. Clin Int Aging. 2015;11:17–27. 10.2147/CIA.S94510 PMC4694673 26730182

[107] Carey RM, Schoeffel CD, Gildea JJ, Jones JE, McGrath HE, Gordon LN, et al. Salt sensitivity of blood pressure is associated with polymorphisms in the sodium-bicarbonate cotransporter. Hypertension. 2012;60:1359–66. 22987918 10.1161/HYPERTENSIONAHA.112.196071 PMC3495588

[108] Williams SM, Ritchie MD, Phillips JA 3rd, Dawson E, Prince M, Dzhura E, et al. Multilocus analysis of hypertension: a hierarchical approach. Hum Hered. 2004;57:28–38. 15133310 10.1159/000077387

[109] Liu C, Xi B. Pooled analyses of the associations of polymorphisms in the GRK4 and EMILIN1 genes with hypertension risk. Int J Med Sci. 2012;9:274–9. 22639547 10.7150/ijms.4171 PMC3360431

[110] Zilbermint M, Hannah-Shmouni F, Stratakis CA. Genetics of hypertension in African Americans and others of African descent. Int J Mol Sci. 2019;20:1081. 30832344 10.3390/ijms20051081 PMC6429313

[111] Bhatnagar V, O’Connor DT, Brophy VH, Schork NJ, Richard E, Salem RM, et al. G-protein-coupled receptor kinase 4 polymorphisms and blood pressure response to metoprolol among African Americans: sex-specificity and interactions. Am J Hypertens. 2009;22:332–8. 19119263 10.1038/ajh.2008.341 PMC2715837

[112] Martinez Cantarin MP, Ertel A, Deloach S, Fortina P, Scott K, Burns TL, et al. Variants in genes involved in functional pathways associated with hypertension in African Americans. Clin Transl Sci. 2010;3:279–86. 21167003 10.1111/j.1752-8062.2010.00242.x PMC5439635

[113] Lohmueller KE, Wong LJ, Mauney MM, Jiang L, Felder RA, Jose PA, et al. Patterns of genetic variation in the hypertension candidate gene GRK4: ethnic variation and haplotype structure. Ann Hum Genet. 2006;70:27–41. 16441255 10.1111/j.1529-8817.2005.00197.x

[114] Staessen JA, Kuznetsova T, Zhang H, Maillard M, Bochud M, Hasenkamp S, et al. Blood pressure and renal sodium handling in relation to genetic variation in the DRD1 promoter and GRK4. Hypertension. 2008;51:1643–50. 18413491 10.1161/HYPERTENSIONAHA.107.109611

[115] Cheng CF, Lin YJ, Lin MC, Liang WM, Chen CC, Chen CH, et al. Genetic risk score constructed from common genetic variants is associated with cardiovascular disease risk in type 2 diabetes mellitus. J Gene Med. 2021;23:e3305. 33350037 10.1002/jgm.3305

[116] Lee M, Kim MK, Kim SM, Park H, Park CG, Park HK. Gender-based differences on the association between salt-sensitive genes and obesity in Korean children aged between 8 and 9 years. PLoS One. 2015;10:e0120111. 25768006 10.1371/journal.pone.0120111 PMC4358955

[117] Zhu H, Lu Y, Wang X, Snieder H, Treiber FA, Harshfield GA, et al. The G protein-coupled receptor kinase 4 gene modulates stress-induced sodium excretion in Black normotensive adolescents. Pediatr Res. 2006;60:440–2. 16940246 10.1203/01.pdr.0000238250.64591.44

[118] Marzano F, Liccardo D, Elia A, Mucio I, de Lucia C, Lucchese AM, et al. Genetic catalytic inactivation of GRK5 impairs cardiac function in mice via dysregulated P53 levels. JACC Basic Transl Sci. 2022;7:366–80. 35540100 10.1016/j.jacbts.2022.01.001 PMC9079799

[119] de Lucia C, Grisanti LA, Borghetti G, Piedepalumbo M, Ibetti J, Lucchese AM, et al. G protein-coupled receptor kinase 5 (GRK5) contributes to impaired cardiac function and immune cell recruitment in post-ischemic heart failure. Cardiovasc Res. 2022;118:169–83. 33560342 10.1093/cvr/cvab044 PMC8752360

[120] Xu B, Li M, Wang Y, Zhao M, Morotti S, Shi Q, et al. GRK5 controls SAP97-dependent cardiotoxic β_1_ adrenergic receptor-CaMKII signaling in heart failure. Circ Res. 2020;127:796–810. 32507058 10.1161/CIRCRESAHA.119.316319 PMC7484403

[121] Ishizaka N, Alexander RW, Laursen JB, Kai H, Fukui T, Oppermann M, et al. G protein-coupled receptor kinase 5 in cultured vascular smooth muscle cells and rat aorta. Regulation by angiotensin II and hypertension. J Biol Chem. 1997;272:32482–8. 9405459 10.1074/jbc.272.51.32482

[122] Keys JR, Zhou RH, Harris DM, Druckman CA, Eckhart AD. Vascular smooth muscle overexpression of G protein-coupled receptor kinase 5 elevates blood pressure, which segregates with sex and is dependent on Gi-mediated signaling. Circulation. 2005;112:1145–53. 16103237 10.1161/CIRCULATIONAHA.104.531657

[123] Sorriento D, Santulli G, Fusco A, Anastasio A, Trimarco B, Iaccarino G. Intracardiac injection of AdGRK5-NT reduces left ventricular hypertrophy by inhibiting NF-kappaB-dependent hypertrophic gene expression. Hypertension. 2010;56:696–704. 20660817 10.1161/HYPERTENSIONAHA.110.155960

[124] Wang L, Shen M, Wang F, Ma L. GRK5 ablation contributes to insulin resistance. Biochem Biophys Res Commun. 2012;429:99–104. 23111327 10.1016/j.bbrc.2012.10.077

[125] da Silva AA, do Carmo JM, Li X, Wang Z, Mouton AJ, Hall JE. Role of hyperinsulinemia and insulin resistance in hypertension: metabolic syndrome revisited. Can J Cardiol. 2020;36:671–82. 32389340 10.1016/j.cjca.2020.02.066 PMC7219403

[126] Lutz SZ, Falcenberg M, Machicao F, Peter A, Kächele M, Randrianarisoa E, et al. Single nucleotide polymorphisms in the G-protein coupled receptor kinase 5 (GRK5) gene are associated with plasma LDL-cholesterol levels in humans. Sci Rep. 2018;8:7745. 29773828 10.1038/s41598-018-26055-7 PMC5958094

[127] Kang S, Hong X, Ruan CW, Yu P, Yu SS, Chen M, et al. Effects of GRK5 and ADRB1 polymorphisms influence on systolic heart failure. J Transl Med. 2015;13:44. 25638254 10.1186/s12967-015-0402-7 PMC4345005

[128] Cresci S, Kelly RJ, Cappola TP, Diwan A, Dries D, Kardia SL, et al. Clinical and genetic modifiers of long-term survival in heart failure. J Am Coll Cardiol. 2009;54:432–44. 19628119 10.1016/j.jacc.2009.05.009 PMC2749467

[129] Stegen M, Frey UH. The role of G protein-coupled receptor kinase 6 regulation in inflammation and pain. Int J Mol Sci. 2022;23:15880. 36555521 10.3390/ijms232415880 PMC9784940

[130] Fraga S, Luo Y, Jose P, Zandi-Nejad K, Mount DB, Soares-da-Silva P. Dopamine D_1_-like receptor-mediated inhibition of Cl/HCO3- exchanger activity in rat intestinal epithelial IEC-6 cells is regulated by G protein-coupled receptor kinase 6 (GRK 6). Cell Physiol Biochem. 2006;18:347–60. 17170521 10.1159/000097612

[131] Fraga S, Jose PA, Soares-da-Silva P. Involvement of G protein-coupled receptor kinase 4 and 6 in rapid desensitization of dopamine D_1_ receptor in rat IEC-6 intestinal epithelial cells. Am J Physiol Regul Integr Comp Physiol. 2004;287:R772–R779. 15166006 10.1152/ajpregu.00208.2004

[132] Rajagopal K, Whalen EJ, Violin JD, Stiber JA, Rosenberg PB, Premont RT, et al. Beta-arrestin2-mediated inotropic effects of the angiotensin II type 1A receptor in isolated cardiac myocytes. Proc Natl Acad Sci USA. 2006;103:16284–9. 17060617 10.1073/pnas.0607583103 PMC1637574

[133] Wang L, Bao H, Wang KX, Zhang P, Yao QP, Chen XH, et al. Secreted miR-27a induced by cyclic stretch modulates the proliferation of endothelial cells in hypertension via GRK6. Sci Rep. 2017;7:41058. 28106155 10.1038/srep41058 PMC5247685

[134] Soliman RH, Pollock DM. Circadian control of sodium and blood pressure regulation. Am J Hypertens. 2021;34:1130–42. 34166494 10.1093/ajh/hpab100 PMC9526808

[135] Palaniappan B, Arthur S, Sundaram VL, Butts M, Sundaram S, Mani K, et al. Inhibition of intestinal villus cell Na/K-ATPase mediates altered glucose and NaCl absorption in obesity-associated diabetes and hypertension. FASEB J. 2019;33:9323–33. 31107610 10.1096/fj.201802673R PMC6662973

[136] Nwia SM, Li XC, Leite APO, Hassan R, Zhuo JL. The Na^+^/H^+^ Exchanger 3 in the intestines and the proximal tubule of the kidney: localization, physiological function, and key roles in angiotensin II-induced hypertension. Front Physiol. 2022;13:861659. 35514347 10.3389/fphys.2022.861659 PMC9062697

[137] Sakr HF, Sirasanagandla SR, Das S, Bima AI, Elsamanoudy AZ. Insulin resistance and hypertension: mechanisms involved and modifying factors for effective glucose control. Biomedicines. 2023;11:2271. 37626767 10.3390/biomedicines11082271 PMC10452601

[138] Varney MJ, Steyaert W, Coucke PJ, Delanghe JR, Uehling DE, Joseph B, et al. G protein-coupled receptor kinase 6 (GRK6) regulates insulin processing and secretion via effects on proinsulin conversion to insulin. J Biol Chem. 2022;298:102421. 36030052 10.1016/j.jbc.2022.102421 PMC9526158

[139] Guo J, Chen H, Ho J, Mancini J, Sontag T, Laporte SA, et al. TGFbeta-induced GRK2 expression attenuates Ang II-regulated vascular smooth muscle cell proliferation and migration. Cell Signal. 2009;21:899–905. 19385060 10.1016/j.cellsig.2009.01.037

[140] Gildea JJ, Tran HT, Van Sciver RE, Bigler Wang D, Carlson JM, Felder RA. A novel role for c-Myc in G protein-coupled receptor kinase 4 (GRK4) transcriptional regulation in human kidney proximal tubule cells. Hypertension. 2013;61:1021–7. 23509080 10.1161/HYPERTENSIONAHA.111.00321 PMC3640476

[141] Yang M, Lin Y, Wang Y, Wang Y. High-glucose induces cardiac myocytes apoptosis through Foxo1/GRK2 signaling pathway. Biochem Biophys Res Commun. 2019;513:154–8. 30952428 10.1016/j.bbrc.2019.03.193

[142] Islam KN, Koch WJ. Involvement of nuclear factor κB (NF-κB) signaling pathway in regulation of cardiac G protein-coupled receptor kinase 5 (GRK5) expression. J Biol Chem. 2012;287:12771–8. 22389501 10.1074/jbc.M111.324566 PMC3339976

[143] Stegen M, Engler A, Ochsenfarth C, Manthey I, Peters J, Siffert W, et al. Characterization of the G protein-coupled receptor kinase 6 promoter reveals a functional CREB binding site. PLoS One. 2021;16:e0247087. 33600497 10.1371/journal.pone.0247087 PMC7891717

[144] Taguchi K, Kobayashi T, Matsumoto T, Kamata K. Dysfunction of endothelium-dependent relaxation to insulin via PKC-mediated GRK2/Akt activation in aortas of ob/ob mice. Am J Physiol Heart Circ Physiol. 2011;301:H571–H583. 21572010 10.1152/ajpheart.01189.2010

[145] Penela P, Ruiz-Gómez A, Castaño JG, Mayor F Jr. Degradation of the G protein-coupled receptor kinase 2 by the proteasome pathway. J Biol Chem. 1998;273:35238–44. 9857063 10.1074/jbc.273.52.35238

[146] Zha Z, Han XR, Smith MD, Lei QY, Guan KL, Xiong Y. Hypertension-associated C825T polymorphism impairs the function of Gβ3 to target GRK2 ubiquitination. Cell Discov. 2016;2:16005. 27462452 10.1038/celldisc.2016.5 PMC4849471

[147] Banday AA, Lokhandwala MF. Oxidative stress reduces renal dopamine D_1_ receptor-Gq/11alpha G protein-phospholipase C signaling involving G protein-coupled receptor kinase 2. Am J Physiol Ren Physiol. 2007;293:F306–F315. 10.1152/ajprenal.00108.2007 17459951

[148] Yu P, Asico LD, Luo Y, Andrews P, Eisner GM, Hopfer U, et al. D_1_ dopamine receptor hyperphosphorylation in renal proximal tubules in hypertension. Kidney Int. 2006;70:1072–9. 16850019 10.1038/sj.ki.5001708

[149] Villar VA, Armando I, Sanada H, Frazer LC, Russo CM, Notario PM, et al. Novel role of sorting nexin 5 in renal D_1_ dopamine receptor trafficking and function: implications for hypertension. FASEB J. 2013;27:1808–19. 23195037 10.1096/fj.12-208439 PMC3633819

[150] Tiu AC, Yang J, Asico LD, Konkalmatt P, Zheng X, Cuevas S, et al. Lipid rafts are required for effective renal D_1_ dopamine receptor function. FASEB J. 2020;34:6999–7017. 32259353 10.1096/fj.201902710RR PMC7200283

[151] Yang J, Villar VAM, Rozyyev S, Jose PA, Zeng C. The emerging role of sorting nexins in cardiovascular diseases. Clin Sci. 2019;133:723–37. 10.1042/CS20190034 PMC6418407 30877150

[152] Okawa T, Aramaki Y, Yamamoto M, Kobayashi T, Fukumoto S, Toyoda Y, et al. Design, synthesis, and evaluation of the highly selective and potent G-protein-coupled receptor kinase 2 (GRK2) inhibitor for the potential treatment of heart failure. J Med Chem. 2017;60:6942–90. 28699740 10.1021/acs.jmedchem.7b00443

[153] Thal DM, Homan KT, Chen J, Wu EK, Hinkle PM, Huang ZM, et al. Paroxetine is a direct inhibitor of g protein-coupled receptor kinase 2 and increases myocardial contractility. ACS Chem Biol. 2012;7:1830–9. 22882301 10.1021/cb3003013 PMC3500392

[154] Sun X, Zhou M, Wen G, Huang Y, Wu J, Peng L, et al. Paroxetine attenuates cardiac hypertrophy via blocking GRK2 and ADRB1 interaction in hypertension. J Am Heart Assoc. 2021;10:e016364. 33372534 10.1161/JAHA.120.016364 PMC7955481

[155] Carotenuto A, Cipolletta E, Gomez-Monterrey I, Sala M, Vernieri E, Limatola A, et al. Design, synthesis and efficacy of novel G protein-coupled receptor kinase 2 inhibitors. Eur J Med Chem. 2013;69:384–92. 24077529 10.1016/j.ejmech.2013.08.039

[156] Ciccarelli M, Sorriento D, Fiordelisi A, Gambardella J, Franco A, Del Giudice C, et al. Pharmacological inhibition of GRK2 improves cardiac metabolism and function in experimental heart failure. ESC Heart Fail. 2020;7:1571–84. 32352228 10.1002/ehf2.12706 PMC7373898

[157] Rainbow RD, Brennan S, Jackson R, Beech AJ, Bengreed A, Waldschmidt HV, et al. Small-molecule G protein-coupled receptor kinase inhibitors attenuate G protein-coupled receptor kinase 2-mediated desensitization of vasoconstrictor-induced arterial contractions. Mol Pharm. 2018;94:1079–91. 10.1124/mol.118.112524 PMC6086822 29980659

[158] Echeverría E, Velez Rueda AJ, Cabrera M, Juritz E, Burghi V, Fabián L, et al. Identification of inhibitors of the RGS homology domain of GRK2 by docking-based virtual screening. Life Sci. 2019;239:116872. 31525427 10.1016/j.lfs.2019.116872

[159] Echeverría E, Ripoll S, Fabián L, Shayo C, Monczor F, Fernández NC. Novel inhibitors of phosphorylation independent activity of GRK2 modulate cAMP signaling. Pharm Res Perspect. 2022;10:e00913. 10.1002/prp2.913 PMC8858223 35184416

[160] Waldschmidt HV, Bouley R, Kirchhoff PD, Lee P, Tesmer JJG, Larsen SD. Utilizing a structure-based docking approach to develop potent G protein-coupled receptor kinase (GRK) 2 and 5 inhibitors. Bioorg Med Chem Lett. 2018;28:1507–15. 29627263 10.1016/j.bmcl.2018.03.082 PMC5916850

[161] Wu Y, Wang S, Wang H, Hu B, Wang J. Selectivity mechanism of GRK2/5 inhibition through in silico investigation. Comput Biol Chem. 2022;101:107786. 36399961 10.1016/j.compbiolchem.2022.107786

[162] Homan KT, Waldschmidt HV, Glukhova A, Cannavo A, Song J, Cheung JY, et al. Crystal structure of G protein-coupled receptor kinase 5 in complex with a rationally designed inhibitor. J Biol Chem. 2015;290:20649–59. 26032411 10.1074/jbc.M115.647370 PMC4543626

[163] Duarte JD, Cavallari LH. Pharmacogenetics to guide cardiovascular drug therapy. Nat Rev Cardiol. 2021;18:649–65. 33953382 10.1038/s41569-021-00549-w PMC8364496

[164] Vandell AG, Lobmeyer MT, Gawronski BE, Langaee TY, Gong Y, Gums JG, et al. G protein receptor kinase 4 (RK4) polymorphisms: β-blocker pharmacogenetics and treatment-related outcomes in hypertension. Hypertension. 2012;60:957–64. 22949529 10.1161/HYPERTENSIONAHA.112.198721 PMC3462355

[165] Sanada H, Yoneda M, Yatabe J, Williams SM, Bartlett J, White MJ, et al. Common variants of the G protein-coupled receptor type 4 are associate ed with human essential hypertension and predict the blood pressure response to angiotensin receptor blockade. Pharmacogenom J. 2016;16:3–9. 10.1038/tpj.2015.6 PMC4559490 25732908

[166] Cao N, Tang H, Tian M, Gong X, Xu Z, Zhou B, et al. Genetic variants of GRK4 influence circadian rhythm of blood pressure and response to candesartan in hypertensive patients. Clin Exp Hypertens. 2021;43:597–603. 33899625 10.1080/10641963.2021.1919357 PMC8565180

[167] Wagner F, Malice MP, Wiegert E, McGrath HE, Gildea J, Mitta S, et al. A comparison of the natriuretic and kaliuretic effects of cicletanine and hydrochlorothiazide in prehypertensive and hypertensive humans. J Hypertens. 2012;30:819–27. 22278145 10.1097/HJH.0b013e32835022a8

[168] Muskalla AM, Suter PM, Saur M, Nowak A, Hersberger M, Krayenbuehl PA. G-protein receptor kinase 4 polymorphism and response to antihypertensive therapy. Clin Chem. 2014;60:1543–8. 25301854 10.1373/clinchem.2014.226605

[169] Rayner B, Ramesar R, Steyn K, Levitt N, Lombard C, Charlton K. G-protein-coupled receptor kinase 4 polymorphisms predict blood pressure response to dietary modification in Black patients with mild-to-moderate hypertension. J Hum Hypertens. 2012;26:334–9. 21544086 10.1038/jhh.2011.33

[170] Xia X, Zeng Y, Li Z, Luo H, Wang W, He Y, et al. Effect of GRK4 on renal gastrin receptor regulation in hypertension. Clin Exp Hypertens. 2023;45:2245580. 37641972 10.1080/10641963.2023.2245580

[171] Wang Y, Li B, Zhao W, Liu P, Zhao Q, Chen S, et al. Association study of G protein-coupled receptor kinase 4 gene variants with essential hypertension in northern Han Chinese. Ann Hum Genet. 2006;70:778–83. 17044852 10.1111/j.1469-1809.2006.00278.x

[172] Montasser ME, Shimmin LC, Gu D, Chen J, Gu C, Kelly TN, et al. Variation in genes that regulate blood pressure are associated with glomerular filtration rate in Chinese. PLoS One. 2014;9:e92468. 24658007 10.1371/journal.pone.0092468 PMC3962404

[173] Frey MK, Dao F, Olvera N, Konner JA, Dickler MN, Levine DA. Genetic predisposition to bevacizumab-induced hypertension. Gynecol Oncol. 2017;147:621–5. 28969913 10.1016/j.ygyno.2017.09.017

[174] Yaqub DA, Moore SC, Rozyyev S, Konkalmatt P, Asico LD, Hunt J, et al. G protein-coupled receptor kinase 4 (GRK4) variant 65L-mediated impairment of dopamine D_1_ receptor function causes hypertension. FASEB J. 2021;35:03664. 10.1096/fasebj.2021.35.S1.03664

[175] Rozyyev S, Konkalmatt P, Villar VA, Asico LD, Kumar M, Hunt J, et al. Molecular mechanisms underlying the GRK4 65L-mediated hypertension in mice. FASEB J. 2019;33:597.2. 10.1096/fasebj.2019.33.1_supplement.597.2

[176] Gainetdinov RR, Bohn LM, Sotnikova TD, Cyr M, Laakso A, Macrae AD, et al. Dopaminergic supersensitivity in G protein-coupled receptor kinase 6-deficient mice. Neuron. 2003;38:291–303. 12718862 10.1016/S0896-6273(03)00192-2

[177] Kavelaars A, Vroon A, Raatgever RP, Fong AM, Premont RT, Patel DD, et al. Increased acute inflammation, leukotriene B4-induced chemotaxis, and signaling in mice deficient for G protein-coupled receptor kinase 6. J Immunol. 2003;171:6128–34. 14634128 10.4049/jimmunol.171.11.6128

